# Pre-miRNA Loop Nucleotides Control the Distinct Activities of *mir-181a-1* and *mir-181c* in Early T Cell Development

**DOI:** 10.1371/journal.pone.0003592

**Published:** 2008-10-31

**Authors:** Gwen Liu, Hyeyoung Min, Sibiao Yue, Chang-Zheng Chen

**Affiliations:** Department of Microbiology and Immunology, Baxter Laboratory of Genetic Pharmacology, Stanford University School of Medicine, Stanford, California, United States of America; University of Arkansas for Medical Sciences, United States of America

## Abstract

**Background:**

Mature miRNAs can often be classified into large families, consisting of members with identical seeds (nucleotides 2 through 7 of the mature miRNAs) and highly homologous ∼21-nucleotide (nt) mature miRNA sequences. However, it is unclear whether members of a miRNA gene family, which encode identical or nearly identical mature miRNAs, are functionally interchangeable *in vivo*.

**Methods and Findings:**

We show that *mir-181a-1*, but not *mir-181c*, can promote CD4 and CD8 double-positive (DP) T cell development when ectopically expressed in thymic progenitor cells. The distinct activities of *mir-181a-1* and *mir-181c* are largely determined by their unique pre-miRNA loop nucleotides—not by the one-nucleotide difference in their mature miRNA sequences. Moreover, the activity of *mir-181a-1* on DP cell development can be quantitatively influenced by nucleotide changes in its pre-miRNA loop region. We find that both the strength and the functional specificity of miRNA genes can be controlled by the pre-miRNA loop nucleotides. Intriguingly, we note that mutations in the pre-miRNA loop regions affect pre-miRNA and mature miRNA processing, but find no consistent correlation between the effects of pre-miRNA loop mutations on the levels of mature miRNAs and the activities of the *mir-181a-1/c* genes.

**Conclusions:**

These results demonstrate that pre-miRNA loop nucleotides play a critical role in controlling the activity of miRNA genes and that members of the same miRNA gene families could have evolved to achieve different activities via alterations in their pre-miRNA loop sequences, while maintaining identical or nearly identical mature miRNA sequences.

## Introduction

A large number of animal miRNAs have been identified and the genes encoding many of these small RNAs have been shown to play diverse functional roles in animals [1]. Each miRNA gene produces at least three small RNA species, including a long primary miRNA transcript (pri-miRNA), an intermediate ∼60-nt precursor miRNA (pre-miRNA), and a ∼21-nt mature miRNA, through sequential endonucleolytic maturation steps [2]. *In vitro* biochemical analyses have indicated that the mature ∼21-nt miRNAs, often the predominant RNA products of miRNA genes, can guide the RNA-induced silencing complex (RISC) to target mRNAs for repression. Pri-miRNA and pre-miRNA are considered to be transitory intermediates during miRNA biogenesis and are thought to play no direct role in gene repression, though they also contain sequences complementary to target element(s) in the 3′untranslated region (UTR) of their cognate target genes.

The deletion or mutation of specific miRNA genes can result in defects in all RNA species produced from the genes [3], [4]. It was known that precursor lin-4 RNA contains the mature lin-4 sequence, and genetic analyses were unable to definitively rule out the possible involvement of precursor lin-4 RNAs in gene regulation [3]. Loss-of-function phenotypes for miRNA genes, such as *lin-4* and *let-7*, were rescued with genomic fragments encoding functional segments of pri-miRNAs, which can be processed into pre-miRNAs and mature miRNAs, but not with the mature miRNA alone [3], [4]. Therefore, the phenotypes observed for loss of miRNA genes cannot be definitively attributed to one of the RNA species made from these genes.

Interestingly, many miRNA genes can be classified into large families consisting of members with highly homologous ∼21-nt mature miRNAs and identical seed nucleotides (6,7), but divergent pre-miRNA loop nucleotides. According to computational and biochemical analyses [5]–[7], the members of a miRNA gene family should regulate a similar set of target genes and have the same biological function. Here we examine whether miRNA genes that encode identical and nearly identical mature miRNAs have the same function. We find that pre-miRNA loop nucleotides play critical roles in controlling the distinct activities of *mir-181a-1* and *mir-181c* genes.

## Results

### Assay for measuring *mir-181a-1* activity in DP cell development

**Figure 1:**
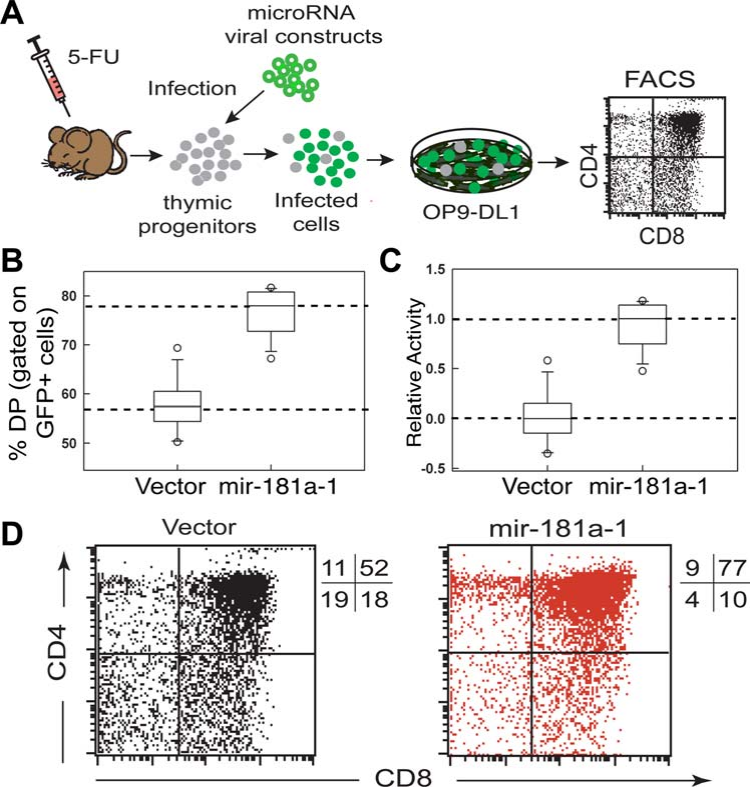
An *in vitro* assay for measuring the activities of miRNA genes on T cell differentiation. (A) Schematics depicting the OP9-DL1 stromal co-culture assay for T cell differentiation. (B) Box-plots to summarize the effects of *mir-181a-1* on the percentage of DP cells differentiated from DN progenitor cells. The results of a representative OP9-DL1 stromal co-culture assay (12 independent replicates for each construct) are shown. (C) Normalized box-plots. The activities of *mir-181a-1* in DP cell development were normalized so that the empty vector (negative control) had a median activity of “0” and *mir-181a-1* expressing vector (positive control) had a median activity of “1.” (D) Representative FACS plots showing the effects of *mir-181a-1* on DP cell development (gated on infected GFP cells).

We used T cell development as a functional readout to determine the nucleotides and structural domains that are required for the function of *mir-181* genes. It is known that *mir-181a-1* plays important roles in T and B lymphocyte development [8]–[10], and can function as a “rheostat” to modulate the strength and threshold of T cell receptor (TCR) signaling [9]. Moreover, mature miR-181a is developmentally regulated during early T cell differentiation in the transition from CD4 and CD8 double-negative (DN) to CD4 and CD8 DP cells in the thymus [9], [10]. Using the OP9-DL1 co-culture assay (Fig. 1A), which can recapitulate the differentiation of DN progenitors into DP cells *in vitro* [11], we showed that ectopic expression of *mir-181a-1* in DN thymic progenitor cells lead to a significant increase in the percentage of DP cells, from a median level of ∼57% in the control group to a median level of ∼77% in the *mir-181a-1* expressing group (Fig. 1B–D). We have found that *mir-181a-1* potentiates DN to DP cell development by targeting negative regulators in the Notch and pre-TCR signaling pathways (Mao T et al., manuscript in preparation). This assay allowed us to quantitatively measure the contribution of nucleotide sequences and structural domains to miRNA gene function via mutagenesis analyses.

### Nucleotides in the pre-miRNA stem region have varied contribution to *mir-181a-1 activity*

**Figure 2:**
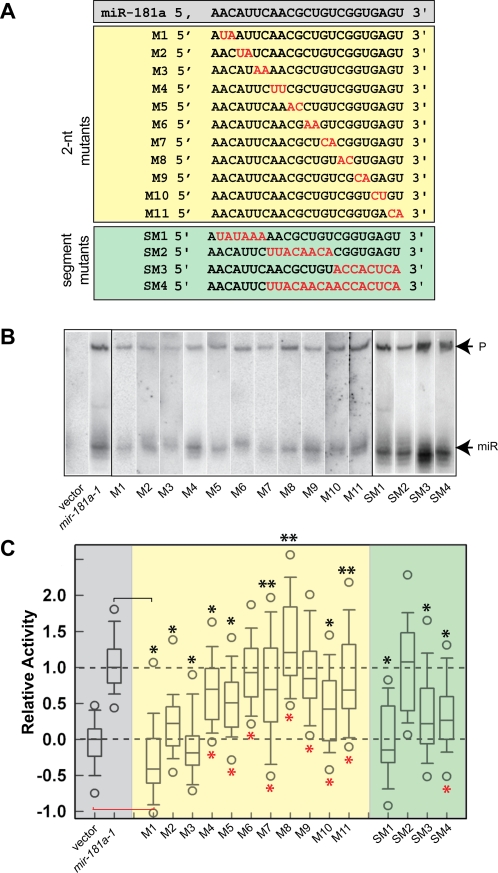
Effects of the mutations in the stem region on *mir-181a-1* activity in promoting DP cell development. (A) Scanning mutations in the stem region of the *mir-181a-1* gene. Two nucleotides (2-nt mutants, shaded yellow) or a stretch of nucleotides (segment mutants, green) in the mature miRNA region are altered (shown in red). Nucleotides are altered to disrupt the potential base pairing to target genes. Compensatory mutations are also generated on the miR* strand to maintain the secondary structure of the pre-miRNAs. (B) Expression and processing of wild-type *mir-181a-1* and stem mutants. Specific probes that perfectly match the mature miR-181a and each of its mutant forms were used in hybridization to determine the expression of mature miR-181a and its stem mutant forms. (C) The effects of *mir-181a-1* and its stem mutants on DP cell development. Normalized data from 3–5 independent T cell assays (each with 12 independent replicates, total 36–60 replicates) are pooled and graphed in the distribution box plots to summarize the distribution of the relative activities of *mir-181a-1* (shaded grey), the 2-nt mutants (shaded yellow), and the segment mutants (shaded green) in DP cell development. Mann-Whitney Rank Sum Tests were performed to determine whether the activities of individual 2-nt mutants were statistically different from those of the control vector (red *, *p*<0.0001) and the *mir-181a-1* vector (black *, *p*<0.0001; **, *p*<0.05) (*Table S1* for statistics). A representative OP9-DL1 stromal co-culture assay without normalization (12 independent replicates for each constructs) is also shown (*Fig. S5*).

To determine which nucleotides of the mature miR-181a region are important for its function, we systematically mutated every set of 2-nt along its 23-nt mature miRNA region (Fig. 2A, yellow). The 2-nt sequences were altered to disrupt potential base pairings to cognate target sequences. To retain the structure of the miRNA stem-loop precursor, we simultaneously mutated the corresponding 2-nt on miR* strand, the complementary strand of the mature miRNA (Fig. S1). Thus, these “stem mutants” contain mutations on both the mature miRNA strands and the miR* strand, affecting the sequences of both pre- and mature miRNA species. Northern analyses of transfected BOSC23 cells demonstrate that the mature miR-181a can be produced from all the mutant constructs (Fig. 2B). The varied intensities of the mature miR-181a and its mutants may not indicate the differences in actual expression levels since different oligo nucleotide probes were used to detect each of the miR-181a mutant forms. When a shorter probe that matches perfectly to both wild-type miR-181a and M1 was used for Northern analyses, we found that the wild-type miR-181a and M1 mature miRNAs were made at comparable levels (Fig. S2).

We then ectopically expressed each of the *mir-181a-1* “stem mutants” in DN thymocytes and examined their effects on DP T cell development using the OP9-DL1 co-culture assay (Fig. 1). By comparing the negative control (empty vector) to the positive control (wild-type *mir-181a-1* expressing vector), it is clear that nucleotides in the stem region have different contributions to *mir-181a-1* activity in promoting T cell development (Fig. 2C, yellow, Table S1 for statistics). The M1 and M3 seed mutants completely abolish *mir-181a-1* activity, while the M2 mutant still display residual activity for promoting T cell development, demonstrating that nucleotides in the seed region play a critical role in the *mir-181a-1* gene function. In comparison, 2-nt mutations outside the seed region have modest effects on *mir-181a-1* activity. The M4, M5, M7, M10, and M11 mutants show a slight reduction in activity. The M6 and M9 mutants have no change in activity, while the M8 mutant show an increase in activity (Table S1 for statistic summary). Thus, nucleotides outside the seed region also contribute to *mir-181a-1* function, but are more tolerant of nucleotide variations.

Since nucleotides outside the 5′ seed region have weaker effects on *mir-181a-1* activity, we then created four additional stem mutants: the segment mutants (SM1–4) with longer stretches of mutations in the *mir-181a-1* stem region (Fig. 2A, green). As shown by Northern blot analyses, these mutants are properly expressed and processed (Fig. 2B). As expected, altering the entire seed region (SM1) completely abolished *mir-181a-1* activity in promoting DP cell development. Interestingly, segment mutants with 3′ 8-nt altered (SM3 and SM4) also exhibit significantly decreased activity, while the mutant with the center 8-nt mutation (SM2) has comparable activity to the wild-type *mir-181a-1* (Fig. 2C, green, Table S1 for statistic summary).

Collectively, these findings demonstrate that the nucleotides in seed region are critical for *mir-181a-1* activity—small alterations in the seed region cause dramatic decreases in activity. In comparison, the nucleotides in the 3′ end of the mature miRNA region have smaller contributions, and the nucleotides in the center of the mature miR-181a have little or no contribution to *mir-181a-1* activity (Fig. 2). These findings confirmed the importance of the seed nucleotides, as shown previously by computational and biochemical analyses [5]–[7], thus validating the use of this assay to measure the activity of *mir-181a-1* genes and to dissect the structural and functional relationships of *mir-181* genes by mutagenesis. However, it is important to note that mutations in stem regions affect mature, precursor, and primary miRNAs, thus it is not possible to attribute the effects of the mutations on DP cell development to one of the RNAs made from the *mir-181a-1* gene.

### *mir-181a-1*, but not *mir-181c*, can promote DP cell development

**Figure 3 pone-0003592-g003:**
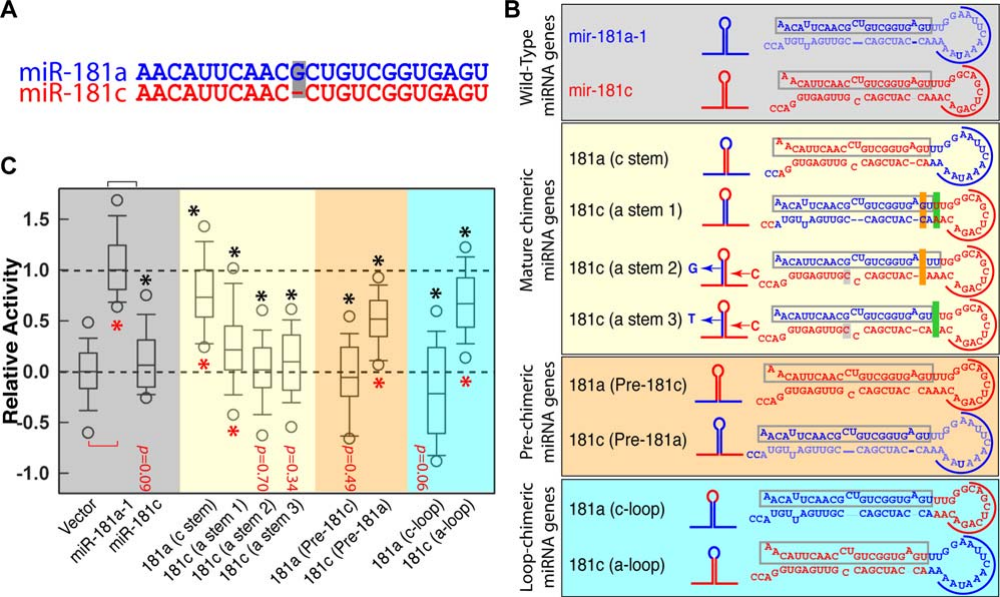
The pre-miRNA loop nucleotides control the distinct activities of *mir-181a-1* and *mir-181c* on DP cell development. (A) Nucleotide sequences of mature miR-181a and miR-181c. (B) Schematics and nucleotide sequences depicting the wild-type *mir-181a-1* and *mir-181c* genes and corresponding precursors (shaded grey). Also shown are the chimeric miRNA genes, with the mature miRNAs, pre-miRNAs, and pre-miRNA loops swapped between *mir-181a-1* and *mir-181c*, termed “mature-chimeric” (shaded yellow), “pre-chimeric” (shaded orange), and “loop-chimeric” (shaded blue), respectively. These mutant genes are designated as *181a(c stem)*, *181c(a stem)*, *181a (pre-181c)*, *181c (pre-181a)*, *181a (c-loop)*, and *181c (a-loop)*. Similar color codes are used in all figures. (C) The effects of the chimeric *mir-181a-1/c* genes on DP cell development. Normalized data from 3–7 independent T cell assays (each with 12 independent replicates for a total of 36–84 replicates) are pooled and graphed in the distribution box plots. Mann-Whitney Rank Sum Tests were performed (*Table S2* for statistics) to determine whether the activities of the chimeric miRNA genes are statistically different from those of the negative control vector (red *, *p*<0.0001) and/or *mir-181a-1* positive control (black *, *p*<0.0001). A representative OP9-DL1 stromal co-culture assay without normalization is also shown (*Fig. S6*).

The members of the *mir-181* family of genes produce four mature miRNAs: miR-181a, miR-181b, miR-181c, and miR-181d, from three putative polycistronic genes, *mir-181a-1/b-1*, *mir-181a-2/b-2*, and *mir-181c/d*, respectively (Fig. S3). The mature miRNAs of the miR-181 family, all of which have identical 5′ seed nucleotides, differ from one another by no more than 3-nt in either the center or the 3′ end of the mature miRNAs. Specifically, mature miR-181a differs from miR-181c by only one nucleotide in the center of the mature miRNA (Figs 3A, 3B). Thus, according to the “seed” hypothesis [5]–[7] and the results of “stem mutant” analyses (Fig. 2), it appears that *mir-181a-1* and *mir-181c* should have similar activities in the co-culture assay.

To test this, we examined the ability of *mir-181a-1* and *mir-181c* to promote DP cell development. Note that mature miR-181a and miR-181c have similar expression patterns during thymocyte development [9], [10], though the endogenous levels of miR-181c is about four to five times lower than that of miR-181a in the corresponding thymocytes, indicating that they are processed in thymocytes and may play roles in normal T cell development (Fig. S4). Thus, the thymocyte differentiation assay allows us to interrogate the functions of *mir-181a-1* and *mir-181c* in a physiologically relevant mRNA and miRNA milieu. Interestingly, while the ectopic expression of *mir-181a-1* results in a substantial increase in the generation of DP cells, the expression of *mir-181c* does not (Fig. 3C, grey), demonstrating that *mir-181a-1* but not *mir-181c* can promote DP cell development (*p*<0.0001, Table S2).

### miRNA genes encoding identical mature miRNAs can have distinct biological activities

To examine whether the single nucleotide difference in the mature miRNA regions contributes to the distinct activities of *mir-181a-1* and *mir-181c*, we generated “mature chimeric” miRNA genes by swapping the stem regions (miR and miR* duplexes) (Fig. 3B, yellow). The resulting “mature chimeric” miRNA genes, termed *181a (c stem)* and *181c (a stem 1)*, should express mature miR-181c and miR-181a, respectively. We also generated two additional “mature chimeric”genes— *181c (a stem 2)* and *181c (a stem 3)*—by replacing mature miR-181c with mature miR-181a while maintaining the miR-181c complementary strand. Even though *181a (c-stem)* is designed to produce mature miR-181c, we observed that this “mature chimeric” miRNA gene was still functionally active in promoting DP cell development, albeit with a median activity of ∼73% of the wild-type *mir-181a-1* (Fig. 3C, yellow, Table S2). In contrast, the *181c (a stem 1)* gene, which encodes mature miR-181a, had a median activity of only ∼21% of the wild-type *mir-181a-1*, and the *181c (a stem 2,3)* genes had no significant activity (Fig. 3C, yellow, Table S2). These results demonstrate that the distinct activities of *mir-181a-1* and *mir-181c* are not caused by the single nucleotide difference between their mature miRNA forms. Notably, these results demonstrate that miRNA genes encoding identical mature miRNAs, such as *mir-181c* and *181a (c-stem)* that encode miR-181c, or *mir-181a-1* and *181c (a-stem1, 2, 3)* that encode miR-181a, can have distinct biological activities.

### Pre-miRNAs and their loops determine the activities of the mir-181 genes

Since *mir-181a-1* and *mir-181c* have divergent pre-miRNA flanking and loop sequences, we then tested whether their differences in activity are determined by their unique pre-miRNAs or by flanking sequences (Fig. 3B). We generated “pre-miRNA chimeric” genes by swapping the pre-miRNA regions between *mir-181a-1* and *mir-181c* (Fig. 3B, orange). When tested in the OP9-DPL1 co-culture assay, the miRNA gene with pre-miR-181a, *181c (pre-181a)*, did promote DP cell development, albeit with a median activity of ∼52% of the wild-type *mir-181a-1*, while the miRNA gene with pre-miR-181c, *181a (pre-181c)*, had no activity (Fig. 3C, orange, Table S2). These results demonstrate that sequences specific to the pre-miRNAs play a key role in determining the distinct biological activities of the *mir-181a-1* and *mir-181c* genes. However, pre-miRNA flanking sequences also contribute to the functions of the *mir-181a-1* and *mir-181c* genes, since the activity of *181c (pre-181a)* is lower than that of the wild-type *mir-181a-1*.

Since pre-miR-181a-1 and pre-miR-181c differ mainly in their pre-miRNA loop nucleotides, we next swapped the pre-miRNA loops and examined the activity of loop chimeras in the OP9-DPL1 co-culture assay (Fig. 3B, blue). We found that *181c (a-loop)* can promote DP cell development with a median activity of ∼67% of the wild-type *mir-181a-1*, while *181a (c-loop)* is inactive in promoting DP cell development (Fig. 3C, blue, Table S2), demonstrating that the distinct biological activities of the *mir-181a-1* and *mir-181c* genes are largely determined by the differences in their pre-miRNA loops.

### The *mir-181a-1 activity is sensitive to nucleotide changes in its pre-miRNA loop*

**Figure 4 pone-0003592-g004:**
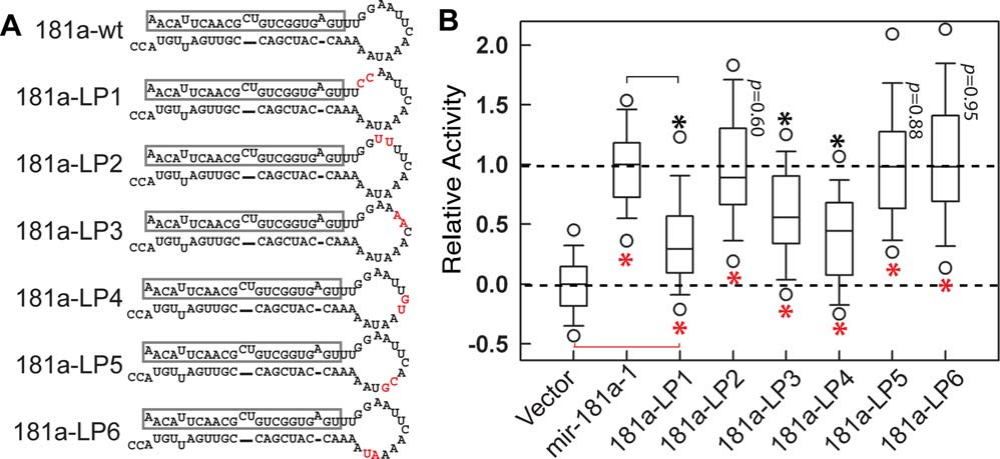
The activity of *mir-181a-1* is sensitive to nucleotide changes in its pre-miRNA loop. (A) Schematics of the pre-miR-181a-1 loop mutants. (B) The effects of pre-miR-181a-1 loop mutants on DP cell development. Normalized data from six independent T cell assays (each with 12 independent replicates for a total of 72 replicates) is shown. Mann-Whitney Rank Sum Tests were performed (*Table S3* for statistics) to determine whether the activities of the loop mutants were statistically different from those of the negative control vector (red *, *p*<0.0001) and/or the *mir-181a-1* positive control vector (black *, *p*<0.0001). A representative OP9-DL1 stromal co-culture assay (12 independent replicates) without normalization is also shown (*Fig. S7*).

To further investigate the role of pre-miRNA loop nucleotides, we carried out scanning mutagenesis around the pre-miR-181a-1 loop (Fig. 4A) and found that dinucleotide mutations in the pre-miR-181a-1 loop had varied effects on *mir-181a-1* activity (Fig. 4B). The *181a-LP1*, *181a-LP3*, and *181a-LP4* mutants had median activities of ∼29%, 55%, and 46% of the wild-type *mir-181a-1*, respectively (Fig. 4B, Table S3). In contrast, the *181a-LP2*, *181a-LP5* and *181a-LP6* mutations did not significantly affect *mir-181a-1* activity. The loop mutagenesis analyses further demonstrated that pre-miRNA loop nucleotides could also quantitatively influence the activity of the *mir-181a-1* gene.

### *mir-181a-1/c* mutants and their wild-type genes produce mature miRNAs with the same 5′ ends

**Figure 5 pone-0003592-g005:**
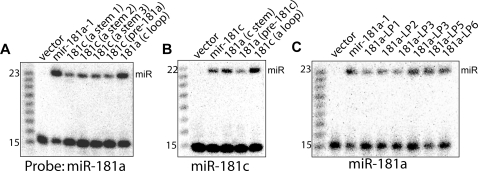
Mature miRNAs produced from the *mir-181a-1/c* mutants have the same 5′ ends. The 5′ ends of mature miR-181a (A) and miR-181c (B) produced from *mir-181a-1/c* domain swapping mutant genes, and the 5′ ends of mature miR-181a (C) produced from *mir-181a-1* loop mutant genes were determined by primer extension analyses. Synthetic miR-181a or miR-181c oligo nucleotides in single nucleotide increments (15 nt–22/23-nt) were radio-labeled and used as size ladders. The upper band represents the major cDNA product of miR-181a (23-nt) or miR-181c (22-nt), and the lower band represents radio-labeled probes for the mature miRNAs.

To understand the mechanisms by which pre-miRNA loop nucleotides may control the activities of miRNA genes, we characterized the effects of pre-miRNA loop mutations on mature miRNA biogenesis. According to computational and biochemical analyses [5]–[7], [12], seed nucleotides are essential for target gene recognition and repression. Since the seed nucleotides are localized at the 5′ end of mature miRNAs and mature miRNA often have polymorphic 3′ or 5′ ends as shown by miRNA cloning analyses [13], [14], a shift of just a few nucleotides in mature miRNA sequences during processing could change the seed nucleotides. To rule out the possibility that mutations in *mir-181a-1/c* cause shifts in the 5′ end of mature miRNAs and changes the seed nucleotides, we carried out primer extension analyses and showed that mature miRNAs produced from various *mir-181a-1/c* mutants have the same 5′ end as those produced from the corresponding wild-type *mir-181a-1/c* genes (Fig. 5A–C). These results demonstrate that *mir-181a-1/c* mutants do not cause changes in the 5′ ends of the mature miRNA sequences, eliminating the possibility that *mir-181a-1/c* mutants affect the activities of the *mir-181a-1* or *mir-181c* genes by controlling the fidelity of the 5′ ends of the mature miRNAs that are produced.

### The effects of *mir-181a-1/c* mutations on the levels of mature miRNA

**Figure 6:**
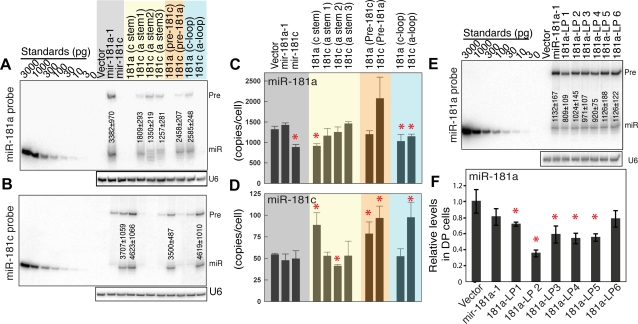
The effects of *mir-181a-1/c* mutants on mature miRNA expression in BOSC 23 and DP cells. (A, B) The copy numbers of mature miR-181a (A) and miR-181c (B) expressed in BOSC 23 cells transfected with the same amounts of vectors expressing different *mir-181a-1/c* mutants determined by quantitative Northern blot analyses (*Fig. S8* for standard curves and quantification, and *Table S4* for statistics). (C, D) The copy numbers of mature miR-181a (C) and miR-181c (D) expressed in DP thymocytes transduced with viral vectors expressing *mir-181a-1/c* mutants determined by miRNA qPCR analyses (*Tables S5* and *S6* for statistics). (E) The copy numbers of mature miR-181a expressed in BOSC 23 cells transfected with the same amounts of viral vectors expressing various *mir-181a-1* loop mutants determined by quantitative Northern blot analyses (*Fig. S9* for standard curves, and *Table S7* for statistics). (F). The copy numbers of mature miR-181a expressed in DP thymocytes transduced with viral vectors expressing various *mir-181a-1* loop mutants, determined by miRNA qPCR analyses (*Table S8* for statistics). Statistical significance was determined by an unpaired two-tailed student's *t* test (compared to the control vector, red *, *p*<0.05). Representative blots of four or more independent quantitative Northern blot analyses are shown (A, B, E).

We then investigated whether *mir-181a-1/c* mutations cause changes in the levels of mature miRNAs made in BOSC 23 and DP cells (Fig. 6), and if so, whether these changes correlate with the effects of loop mutations on the activities of miRNA genes. BOSC 23 cells do not express endogenous mature miR-181a or miR-181c, thus facilitating accurate measurement of mature miRNA levels produced from the mutant constructs. Quantitative Northern blot analyses were used to define the levels of mature miR-181a and miR-181c, as well as the sizes of the mature miRNAs and the levels of the pre-miRNAs produced from various *mir-181a-1/c* mutant constructs in BOSC 23 cells (Fig. 6A, 6B, 6E, and S8). Since it is difficult to obtain sufficient numbers of infected DP thymocytes for Northern blot analyses, we carried out miRNA qPCR analyses to determine the number of copies of mature miR-181a and miR-181c in DP cells transduced with *mir-181a-1/c* mutant viruses (Fig. 6C, 6D, 6F). Comparing the effects of *mir-181a-1/c* mutations on the levels of mature miRNA in two different cell types may also reveal whether *mir-181a-1/c* loop mutations cause differential miRNA processing in different cell types.

We find that mature miR-181a levels in either DP or BOSC 23 cells expressing the *mir-181a-1/c* mutants have no apparent correlation to the activities of mutant genes (Figs 3, 4, 6, S8, and S9). Ectopic expression of *mir-181a-1* and *mir-181c* results in comparable levels of mature miR-181a and miR-181c in BOSC 23 cells (Figs. 6A, 6B, and S8), but not significant increases in mature miR-181a or miR-181c levels in the infected DP cells (Fig. 6C, 6D, 6F). Since *mir-181a-1* activity can be effectively abolished by mutations in the seed region (Fig. 2), these findings suggest that we have probably measured the activity of the *mir-181a-1* gene in T cell development, and that *mir-181a-1* may exert its biological function without a significant increase in the mature miRNA level (Fig. 2, 3, 4, 6C). Nevertheless, we cannot rule out that a small but undetectable increase in the mature miR-181a level is sufficient for *mir-181a-1* activity. In another case, the *181a-LP2* mutant exhibited the same activity as wild-type *mir-181a-1*, but DP cells infected with this mutant expressed ∼50% less mature miR-181a (Figs. 4B, 6E, 6F). Finally, *181a-LP3*, *181a-LP4*, and *181a-LP5* produced similar levels of mature miR-181a in DP cells and BOSC 23 cells (Fig. 6E, 6F, and S9), but these mutants had distinct activities in promoting DP cell development (Fig. 4B). The *181a-LP5* mutations had no effect on *mir-181a-1* activity, while *181a-LP3* and *181a-LP4* mutations caused a ∼45% and 56% reduction in median activity, respectively.

In comparison, the levels of mature miR-181c in DP cells expressing *mir-181a-1/c* mutants also have no consistent correlation to their activities in DP cell development (Fig. 3, 6D). Ectopic expression of *181a (c-stem)*, *181a (pre-181c)*, and *181c (a-loop)* resulted in significant increases in the levels of mature miR-181c in DP cells (Fig. 6D). However, while *181a (c-stem)* and *181c (a-loop)* can promote DP cell development, *181a (pre-181c)* cannot (Fig. 3C), showing that increases in mature miR-181c in DP cells also do not always correlate with the activities of corresponding miRNA genes. Interestingly, mature miRNA expression from some miRNA genes might be differentially regulated in DP and BOSC 23 cells, but such differential regulation in mature miRNA processing also has little correlation to the activity of miRNA genes. For example, while *mir-181c, 181a (c-stem)*, *181a (pre-181c)*, and *181c (a-loop)* all produce similar levels of mature miR-181c in BOSC 23 cells (Figs. 6B and S8), ectopic expression of *181a (c-stem)*, *181a (pre-181c)*, and *181c (a-loop)*, but not *mir-181c*, resulted in significant increases in the levels of mature miR-181c in DP cells (Fig. 6D). However, among the ones that caused increases in mature miR-181c levels in DP cells, *181a (c-stem)* and *181c (a-loop)* can promote DP cell development, while *181a (pre-181c)* cannot. Collectively, above analyses show that usually mature miR-181a and miR-181c levels in DP cells or BOSC 23 cells expressing the *mir-181a-1/c* and their mutants were the same within a factor of two, and in cases where different RNA levels were found, there was no consistent correlation with the activities of corresponding miRNA genes (Figs 3, 4, 6, S8, S9).

## Discussion

**Figure 7:**
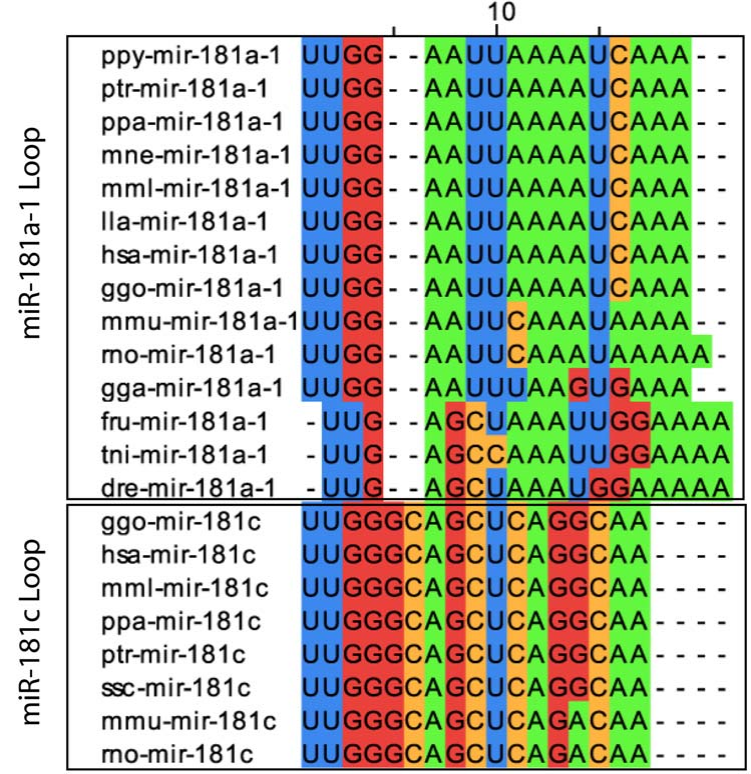
Phylogenetic comparison of pre-miR-181a-1 and pre-miR-181c loop sequences. The full genus and species names and their abbreviations are as follows: Danio rerio, dre; Fugu rubripes, fru; Homo sapiens, hsa; Gallus gallus, gga; Gorilla gorilla, ggo; Lagothrix lagotricha, lla; Macaca mulatta, mml; Mus musculus, mmu; Macaca nemestrina, mne; Pan paniscus, ppa; Pongo pygmaeus, ppy; Pan troglodytes, ptr; Rattus norvegicus, rno; Sus scrofa, ssc; Tetraodon nigroviridis, tni.

We have identified the nucleotide sequences and structural domains that are required for the function of *mir-181a-1 and mir-181c* through mutagenesis and domain-swapping analyses. We show that not only the nucleotides in the 5′ and 3′ ends of the stem but also those in the pre-miRNA loop are critical for *mir-181a-1* activity. We find that *mir-181a-1* and *mir-181c* have distinct activities in early T cell development, and the distinct activities of *mir-181a-1* and *mir-181c* are controlled by their pre-miRNA loops (Fig. 3, 4), indicating that miRNA genes encoding identical or nearly identical mature miRNAs can exert different biological activities determined by their unique loop nucleotides. Interestingly, the pre-miRNA loop sequences of *mir-181a-1* and *mir-181c* are divergent, but each is evolutionarily conserved in multiple animal species (Fig. 7), suggesting that members of the same miRNA gene families may have evolved to achieve distinct specificities or degrees of activity via alterations in their pre-miRNA loop sequences. However, *mir-181a-1/c* mutants do not change the 5′ ends of mature miRNAs produced (Fig. 5) and the levels of mature miRNAs produced from these genes have no consistent correlation with the activities of corresponding miRNA genes (Fig. 6). Clearly, our results demonstrate that pre-miRNA loop nucleotides have a key role in controlling miRNA gene function.

When interpreting the above findings, it is critical to draw a distinction between the activity of a miRNA gene and the activity of a mature miRNA, which are often used interchangeably in the literature. In this study, we have examined the activities of miRNA genes, which may be contributed by one or more of the RNAs (mature miRNAs and their precursors) made from these miRNA genes, not the activities of mature miRNAs alone. Although siRNA duplexes have been shown to function as miRNA surrogates to target gene repression when transfected into mammalian cells [7], mature miRNAs delivered into cultured cells by transfection are diluted quickly during cell expansion, prohibiting their use in long-term T cell development culture assays. Thus, we were unable to quantitatively measure whether transfected mature miRNAs might be functionally equivalent to RNAs produced from miRNA genes. Furthermore, given the complex small RNA sorting pathways in animal cells [15], [16], we have not attempted to determine the efficiency of transfected miRNAs being incorporating into the pathways used by corresponding miRNA genes *in vivo*.

It is also important to emphasize that limited conclusions can be drawn by correlating the changes in mature miRNA levels to the activities of corresponding mutant miRNA genes (Fig. 3, 4, 6). We believe that such analyses can only establish correlations, but not causal relationships, between the levels of mature miRNA expression and the activity of miRNA genes. However, strong and positive correlations would support that pre-miRNA loop nucleotides control the activity of miRNA genes by influencing mature miRNA levels, whereas negative or inconsistent correlations would disfavor such idea. Most importantly, miRNA genes make at least three RNA species, including pri-miRNA, pre-miRNA, or mature miRNA, prohibiting us from definitively attributing miRNA gene functions to one of these RNAs in the T cell assays. Moreover, the complex regulatory steps in the miRNA biogenesis pathways and during early T cell development may also compromise the robustness of such correlation analyses. Finally, we cannot determine whether alterations in pre-miRNA loop sequences affect all or only selected cognate targets. Since multi-target regulation is required for the *mir-181a-1* function in T cells and the ectopic expression of a single miR-181a insensitive target is sufficient to block the function of the *mir-181a-1* [9], it is possible that pre-miRNA loop nucleotides only contribute to the regulation of one or a few targets among those that are regulated by the *mir-181a/c* or their mutants.

**Figure 8:**
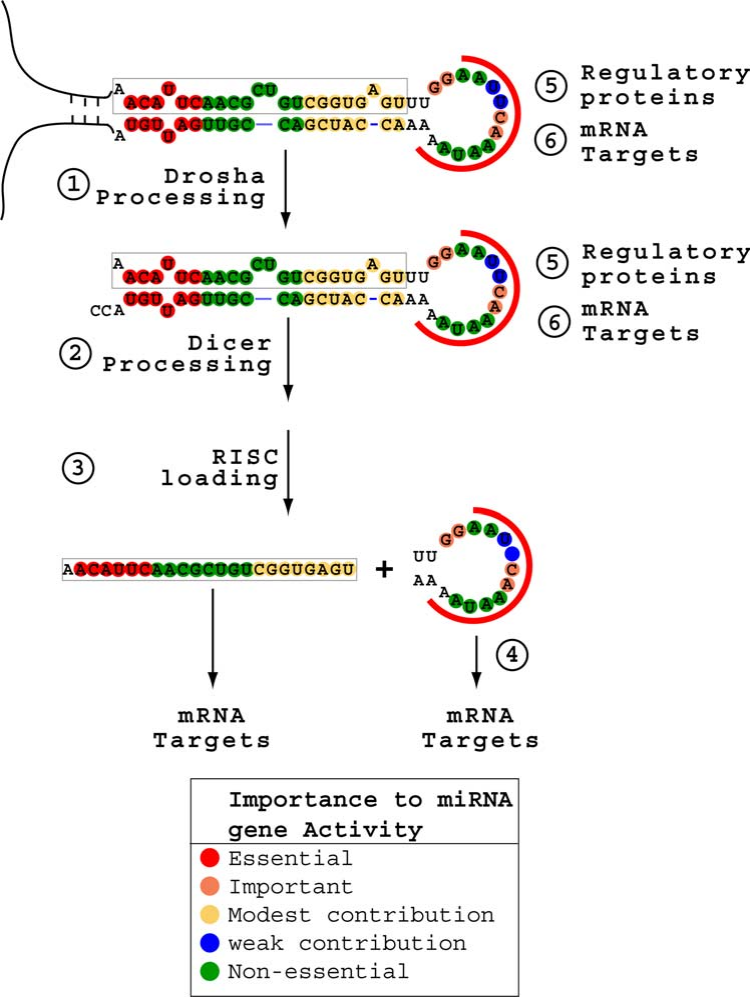
A “heat map” of the functionally important nucleotides in the pre-miR-181a-1 region according to mutagenesis analyses. Color-code was used to illustrate the importance of the pre-miRNA nucleotides to the activity of the *miR-181a-1* gene. Possible mechanisms by which pre-miRNA loop nucleotides control the activities of miRNA genes are listed.

Nevertheless, based on the functional importance of various nucleotides in *mir-181a-1* genes, we may postulate some mechanisms by which pre-miRNA loop nucleotides control the activities of miRNA genes (Fig. 8). Pre-miRNA loops might influence miRNA function by controlling the processing of pri-miRNAs into pre-miRNAs, the transport and sub-cellular localization of pre-miRNAs, the processing of pre-miRNAs into mature miRNAs, or the loading of mature miRNAs into RISCs [15]–[21]. For example, animal cells are thought to have multiple RISCs with distinct Argonatue proteins (AGO1–4). If pre-miRNA loops play a role in guiding small RNAs into various RISCs, which might have different efficiencies in gene silencing [15], [16], then mature miRNA levels would not necessarily correlate with the function of the miRNA genes. Moreover, functional miRNAs may be generated from pre-miRNA loops, since pre-miRNA loop-derived small RNAs have been found in miRNA cloning and deep sequencing analyses [22]. Finally, recent studies have shown that LIN-28 may recognize the pre-let-7 loop nucleotides and block the processing of human pri-let-7 RNAs into mature miRNAs in embryonic stem cells, suggesting that pre-miRNA loops may be recognized by LIN-28 or LIN-28-like RNA binding factors that control mature miRNA biogenesis [23]–[25]. However, these RNA binding factors would control the activity of miRNA genes by blocking mature miRNA biogenesis [23]–[25], which is inconsistent with the fact that the levels of mature miR-181a/c have no consistent correlations with the effects of corresponding mutations on the activities of the *mir-181a-1/c* genes (Figs 3, 4, 6). Therefore, pre-miRNA loop nucleotides may not control *mir-181a-1/c* activity through recognizing the LIN-28 or LIN-28-like RNA binding factors.

Since pre-miRNA loop nucleotides — not the levels of mature miRNAs — were more critically linked to the function of miRNA genes, our findings raised an important question: which of the RNA species synthesized from miRNA genes is the functional one(s)? Definitively addressing this question is vital to understanding of the mechanisms by which miRNA genes control gene expression. One possibility is that pri-miRNAs and/or pre-miRNAs may have functional roles in target gene regulation before they are further processed. Supporting this idea, our findings demonstrate that pri-miRNAs and pre-miRNAs contain not only the mature miRNA sequences that can pair with cognate target sites, but also the loop nucleotides that are important for the activity and functional specificity of miRNA genes. Moreover, pre-miRNA-like stem-loop structures have been shown to be a common module for intermolecular RNA:RNA interactions [26], [27]. Thus, based on these findings (Figs 2–6), we speculate that pre-miRNA loops may play roles in target gene regulation as a functional motif of the pri-miRNA and pre-miRNA RNAs. This model can explain why both pre-miRNA loop and “seed” nucleotides play key roles with respect to the function of corresponding wild-type and mutant *mir-181a-1/c* genes.

## Materials and Methods

### Retroviral constructs for miRNA gene expression

A double-copy retroviral vector with a human H1 polymerase III expression cassette was used to express *mir-181a-1*, *mir-181c*, and their mutant genes. Briefly, a ∼270-nt gene segment containing a ∼22-nt mature miRNA and ∼125-nt of genomic sequences flanking both sides of the miRNA was amplified from genomic DNA and placed in the U3 region of the 3′ LTR under the control of the human H1 pol III promoter [8], [9]. A GFP reporter driven by an independent murine 3-phosphoglycerate kinase promoter (P_PGK_) was used as a marker for infection. *mir-181a-1* and *mir-181c* mutant constructs were generated using an overlapping PCR strategy to introduce mutations in the stem and loop regions of the miRNA genes. All mutant constructs were validated by DNA sequencing (see Materials and Methods S1 for the wild-type and mutant gene sequences). For mutations in the miRNA stem regions, compensatory mutations were also introduced to the miR* strands to preserve the integrity of the stem and loop structures (Fig. S1). High-titer retroviral supernatant was generated by co-transfecting the miRNA expression vector and pCLeco packaging construct into BOSC 23 cells (293T-based viral packaging cell line).

### OP9-DL1 stromal co-culture assay for *in vitro* T cell differentiation

Six-week-old male C57BL/6J mice were obtained from the Jackson Laboratory (Bar Harbor, ME). The mice were given a single intravenous dose of 5-fluorouracil (5-FU; 150 mg/kg body weight; SIGMA, St. Louis, Missouri) four days before culture initiation. All mice were maintained at the Stanford University School of Medicine animal facility and treated in accordance with the Stanford University Laboratory Animal Care guidelines. Thymocytes were isolated from the 5-FU primed-mice, infected with miRNA expression vectors by spinoculation, and seeded at 1×10^5^ infected cells/well into 24-well tissue culture plates containing a monolayer of OP9-DL1 stromal cells. For each viral construct, 12 independent culture replicates were seeded. The cells were cultured in Minimum Essential Medium (MEM) Alpha Medium supplemented with 20% FCS, 10 mM Hepes, 1 mM Sodium pyruvate, 5 ng/ml IL-7, and 27.5 ng/ml Flk2/Flt3L for 24 hours and then the medium was changed to remove non-adherent thymocytes. The cultures were fed with fresh medium on day 6. After about 8–10 days of culturing, cells were harvested and stained for surface marker CD4, CD8, and CD45. The percentage of DP cells yielded from culture was quantified by flow cytometry. Both adherent and non-adherent cells were collected. Adherent cells were dissociated by treatment with collagenase type IV (0.8 mg/ml; Worthington, Lakewood, NJ) followed by forceful pipetting. The cells were then immunolabeled with PE-conjugated anti-CD4 antibody (clone RM4-5; BD Pharmingen, San Diego, CA) and PE-Cy5-conjugated anti-CD8a antibody (clone 53-6.7; BD Pharmingen) and analyzed on a FACSCalibur (BD Biosciences, San Jose, CA) for the expression of CD4 and CD8 cell surface antigens. GFP positive thymocytes were distinguishable from GFP positive stromal cells by forward and side scatter gates and the intensity of green fluorescence. When the infection-rate was low, anti-CD45 antibody staining was used to gate out contaminating GFP+ OP9-DL1 cells. The appropriate dilution for each antibody was determined prior to use.

Box-plots summarize the distribution of relative miRNA activity in DP cell development. The ends of the boxes define the 25^th^ and 75^th^ percentiles, a line indicates the median, and bars define the 5^th^ and 95^th^ percentiles. Individual outliers are also shown. The activities of *mir-181a-1*, *mir-181c*, and mutant genes in DP cell development were normalized so that the empty vector (negative control) had a median activity of “0” and *mir-181a-1* expressing vector (positive control) had a median activity of “1.” The percentage of DP cells yielded from the co-culture assay varied between experiments, possibly due to the heterogeneous nature of the thymic progenitor cells and intrinsic variation between the batches of mice used. Therefore, such normalization is required to reset the baseline and allows the independent repeats to be compared. Mann-Whitney Rank Sum Tests were performed to determine whether the activities of mutants were statistically different from the control vector or the *mir-181a-1* vector.

### Cell culture and transfection

Adherent BOSC 23 cells were grown in DMEM, 10% FBS, 1% of pen/strep antibiotics, supplemented with glutamine. For northern and primer extension assays, BOSC 23 cells were plated at a density of 3.75×10^5^ cells/well in a 6-well plate at 24 hours before transfection. Cells were transfected with 2 µg constructs expressing *mir-181a-1*, *mir-181c*, mutant genes, and control vector using Fugene transfection reagents (Roche).

### Quantitative Northern blot analyses

Quantitative Northern blot analyses were used to determine the level of mature miRNA expression and processing of the pre-miRNA and mature miRNA. Total RNA was prepared from BOSC 23 cells transfected with constructs expressing *mir-181a-1* or *mir-181c*, and their mutant genes, and loaded onto 15% PAGE gel (10 µg/sample). Since all of the miRNA expressing vectors contain an independent GFP reporter, the percentage of GFP positive cells were determined by FACS analyses and used to control for variations in transfection efficiency. Various amounts of synthetic mature miRNA were loaded onto the same gel to generate standard curves. Specific probes that perfectly matched mature miRNAs were used in hybridization to determine the expression of both mature and pre-miRNA species (see Materials and Methods S1 for probes sequences). Band intensity was determined by phosphoimager quantification. Blots were also probed with U6 probes to normalize loading. Exact copies/pg of total RNA were determined by comparing to the corresponding standard curve. Representative blots of three or more independent quantitative Northern blot analyses were shown. Standard curves and average results of three or more independent quantitative Northern blot analyses were summarized and plotted.

### Primer Extension Analyses

Primer extension was used to map the 5′ ends of the mature miRNAs produced from the *mir-181a-1/c* mutant genes. Total RNA was prepared from BOSC 23 cells 48 hours after transfection with constructs expressing *mir-181a-1*, *mir-181c*, and their mutant genes. P^32^ labeled primer was mixed with appropriate RNA samples (10 µg total RNA) in the reaction buffer (1×RT reaction buffer with 0.25 mM of each dNTP), heated at 55°C for 20 minutes, and slowly cooled to 16°C to allow for annealing. The primer extension reaction was initiated by adding reverse transcriptase and then carried out at 16°C for 20 minutes, 42°C for 2 hours, and 85°C for 5 minutes. Samples were then resolved on a 15% denaturing PAGE gel. Synthetic miR-181a or miR-181c oligos in single nucleotide increments (15 nt–22/23 nt) were labeled and loaded onto the gel as size ladder. The primer extension results were visualized by overnight exposure to a phosphoimager screen (see Materials and Methods S1 for probes and DNA ladder sequences).

### miRNA qPCR analyses

In the OP9-DL1 culture assay, mixed DN (CD4^−^ CD8^−^) thymic progenitor cells differentiate into DP cells though multiple stages that are characterized by unique cell surface markers and complex molecular events. The DP cell population in the OP9-DL1 culture represents a relatively homogeneous population and can be isolated by FACS-sorting in reasonable quantity for miRNA qPCR analyses. In brief, GFP positive DP cells from OP9-DL1 co-culture assay were isolated by FACS-sorting (>94% pure). Synthetic miR-223 was spiked into sorted cells at the ratio of 100 pmol of miR-223 per 100,000 cells before RNA purification. Total RNA was isolated using Trizol reagent (Invitrogen, Carlsbad, CA). We assumed that the ratio of spiked miR-223 to a miRNA of interest would not change during RNA purification. cDNA was then synthesized using miRNA-specific looped primers (Applied Biosystems, Foster city, CA) and amplified with miRNA-specific forward primers, a TaqMan probe, and reverse primers (Applied Biosystems). PCR amplification was performed in triplicate in an ABI-7000 sequence detection system (Applied Biosystems) at 95°C for 10 minutes followed by 40 cycles at 95°C for 15 seconds and 60°C for 1 minute. To determine the exact copy number of a miRNA in sorted DP cells, we carried out absolute miRNA quantification with miRNA qPCR assay. Exact copies of test and spiked miRNAs in the defined amount of total RNA input were determined by using standard curves for mature miR-181a, miR-181c, and spiked miR-223. miR-181a or miR-181c expression was normalized using miR-15b as an endogenous loading control. Representative results of three miRNA qPCR analyses of independently-sorted, virally infected DP cells were shown. All reactions were carried out according to the manufacturer's instructions.

## Supporting Information

Materials and Methods S1 (0.03 MB DOC)

Figure S1 Schematics and nucleotide sequences depict mature *mir-181a-1* mutants. Compensatory mutations are introduced to maintain the integrity of the pre-miRNA secondary structure.
(0.95 MB TIF)

Figure S2 Expression and processing of wild-type *mir-181a-1* and the M1 stem mutant gene. (A) Nucleotide sequences of the wild-type miR-181a and the M1 mutant. (B) Northern blot analyses of mature miRNA expression from the wild-type miR-181a and the M1 mutant. Total RNA was prepared from BOSC cells transfected with constructs expressing *mir-181a-1*, or the M1 mutant genes. Relative transfection efficiencies were determined by qPCR analyses of GFP mRNA levels produced from the transfected miRNA constructs, then used to normalize RNA loadings in Northern blot analyses. A shorter probe that perfectly matches to both mature miR-181a and the M1 mutant forms is used in hybridization to determine the expression of mature miR-181a and its mutant forms. Relative expression levels of the mature miRNAs determined by phosphoimager quantification is indicated.
(1.46 MB TIF)

Figure S3 Members of the *mir-181* gene family. (A) Alignment of the mature miR-181 miRNAs. (B) Schematics and nucleotide sequences depicting the pre-miRNAs of the human and mouse *mir-181* gene family members.
(0.98 MB TIF)

Figure S4 Developmental regulation of miR-181c expression in various purified thymocyte populations determined by miRNA qPCR.
(0.23 MB TIF)

Figure S5 The effects of mutations in the mature miRNA region of the *mir-181a-1* genes on DP cell development (SI Fig. 1). (A) Box-plots summarize the percent of DP cells generated from DN progenitor cells infected with *mir-181a-1*, or mature miRNA mutant genes (gated on GFP positive). A representative OP9-DL1 stromal co-culture assay (12 independent replicates for each construct) is shown. The ends of the boxes define the 25^th^ and 75^th^ percentiles, a line indicates the median, and bars define the 5^th^ and 95^th^ percentiles. (B) Statistical summary. Mann-Whitney Rank Sum Tests were performed on this representative data set to determine whether the activity of *mir-181a-1*, *mir-181c*, or their chimeric mutants is statistically different from the control vector or the *mir-181a-1* vector.
(2.34 MB TIF)

Figure S6 The effects of the chimeric *mir-181a-1* and *mir-181c* genes on DP cell development (Fig. 2). (A) Box-plots summarize the percent of DP cells generated from DN progenitor cells infected with *mir-181a-1*, *mir-181c*, or their chimeric mutants (GFP positive). A representative OP9-DL1 stromal co-culture assay (12 independent replicates for each construct) is shown. The ends of the boxes define the 25^th^ and 75^th^ percentiles, a line indicates the median, and bars define the 5^th^ and 95^th^ percentiles. (B) Statistical summary. Mann-Whitney Rank Sum Tests were performed on this representative data set to determine whether the activity of *mir-181a-1*, *mir-181c*, or their chimeric mutants is statistically different from the control vector or the *mir-181a-1* vector.
(1.92 MB TIF)

Figure S7 The effects of the pre-miR-181a-1 loop mutants on DP cell development (Fig. 3). (A) Box-plots summarize the percent of DP cells generated from DN progenitor cells infected with *mir-181a-1*, or pre-miR-181a-1 loop mutant genes (GFP positive). A representative OP9-DL1 stromal co-culture assay (12 independent replicates for each construct) is shown. The ends of the boxes define the 25^th^ and 75^th^ percentiles, a line indicates the median, and bars define the 5^th^ and 95^th^ percentiles. (B) Statistical summary. Mann-Whitney Rank Sum Tests are performed on this representative data set to determine whether the activity of *mir-181a-1* and pre-miRNA loop mutant genes is statistically different from the empty vector (negative control) or the *mir-181a-1* expressing vector (positive control).
(1.07 MB TIF)

Figure S8 Mature and pre-miRNA expression levels from the chimeric *mir-181a-1* and *mir-181c* genes (Fig. 5A, B). Total RNA was prepared from BOSC cells transfected with constructs expressing *mir-181a-1*, *mir-181c*, and the chimeric *mir-181a-1* and *mir-181c* genes. Since all miRNA vectors contain an independent GFP reporter, percentage cells that are GFP positive were determined by FACS analyses and used to control for variations in transfection efficiency. Quantitative Northern blot analyses were carried out to determine the expression of *mir-181a-1*, *mir-181c*, and the chimeric *mir-181a-1* and *mir-181c* genes. Specific probes that perfectly match to mature miR-181a or miR-181c were used in hybridization to determine the expression of mature and pre-miRNA forms. Band intensities were determined by phosphoimager quantification and normalized to the levels of wild-type controls accordingly. (A) Standard Curves for miR-181a and miR-181c. (B) The copies of mature miR-181a and miR-181c in BOSC 23 cells transfected with *mir-181a-1/c* mutants determined by quantitative Northern blot analyses. Average results of four independent experiments were plotted (*Table S4* for statistics). (C) Relative levels of pre-miR-181a and pre-miR-181c in BOSC 23 cells transfected with *mir-181a-1/c* mutants determined by Northern blot analyses. Average results of four independent experiments were plotted.
(2.36 MB TIF)

Figure S9 Mature and pre-miRNA expression levels from the pre-miR-181a-1 loop mutant genes (Fig. 5E). Total RNA was prepared from BOSC cells transfected with constructs expressing the *mir-181a-1* loop mutant genes. Since all miRNA vectors contain an independent GFP reporter, percentage cells that are GFP positive were determined by FACS analyses and used to control for variations in transfection efficiency. Quantitative Northern blot analyses were carried out to determine the expression of *the pre-mir-181a-1* loop mutant genes. A probe that perfectly matches to the mature miR-181a was used in hybridization to determine the expression of mature and pre-miRNA forms. Band intensity was determined by phosphoimager quantification and normalized to the levels of wild-type controls accordingly. (A) Standard Curves for miR-181a. (B) The copies of mature miR-181a in BOSC 23 cells transfected with *mir-181a-1* loop mutants determined by quantitative Northern blot analyses. Average results of four independent experiments were plotted (*Table S7* for statistics). (C) Relative levels of pre-miR-181a in BOSC 23 cells transfected with *mir-181a-1* loop mutants determined by Northern blot analyses. Average results of four independent experiments were plotted.
(0.92 MB TIF)

Table S1 Summary of the statistical analyses on the activity of *mir-181a-1* genes with mutations in the mature miRNA region. The activities of the wild-type *mir-181a-1* and its variants with mutations in the mature miRNA regions in promoting DP cell development are normalized so that the empty vector (negative control) has a median activity of “0” and the wild-type *mir-181a-1* vector (positive control) has a median activity of “1.” Normalized data from 3–5 independent T cell assays (each with 12 independent replicates, total 36–60 replicates) are pooled and graphed in the distribution box plots. Mann-Whitney Rank Sum Tests are performed to determine whether the activity of *mir-181a-1* and mature miRNA mutant genes is statistically different from the empty vector (negative control) or the *mir-181a-1* expressing vector (positive control).
(0.05 MB DOC)

Table S2 Summary of the statistical analyses on the activity of the *mir-181a-1* and *mir-181c* mutant genes. The activities of *mir-181a-1*, *mir-181c*, and mutant genes in promoting DP cell development are normalized so that the empty vector (negative control) has a median activity of “0” and the *mir-181a-1* expressing vector (positive control) has a median activity of “1.” Normalized data from 3–7 independent T cell assays (each with 12 independent replicates, total 36–84 replicates) are pooled and graphed in the distribution box plots. Mann-Whitney Rank Sum Tests are performed on the pooled data set to determine whether the activity of *mir-181a-1* and *mir-181c* mutant genes is statistically different from the empty vector (negative control) or the *mir-181a-1* expressing vector (positive control).
(0.04 MB DOC)

Table S3 Summary of the statistical analyses on the activity of the *mir-181a-1* genes and mutants with nucleotides in the pre-miRNA loop region altered. The activity of *mir-181a-1* and its pre-miRNA loop mutant genes in promoting DP cell development are normalized so that the empty vector (negative control) has a median activity of “0” and the *mir-181a-1* expressing vector (positive control) has a median activity of “1.” Normalized data from at least 6 independent T cell assays (each with 12 independent replicates, total 72 replicates) are pooled and graphed in the distribution box plots. Mann-Whitney Rank Sum Tests are performed on the pooled data set to determine whether the activity of *mir-181a-1* and pre-miRNA loop mutant genes is statistically different from the empty vector (negative control) or the *mir-181a-1* expressing vector (positive control).
(0.03 MB DOC)

Table S4 Summary of the statistical analyses on the mature *miR-181a* and *miR-181c* levels in transfected BOSC cells. The copy numbers of mature *miR-181a* and *miR-181c* expressed in BOSC cells transfected with constructs expressing *mir-181a-1*, *mir-181c*, and the chimeric *mir-181a-1* and *mir-181c* genes were determined by quantitative Northern blot. Statistical significance was determined by analyzing the results of four independent quantitative Northern blot analyses using an unpaired two-tailed student's t test.
(0.03 MB DOC)

Table S5 Summary of the statistical analyses on the mature *miR-181a* levels in infected DP T cells. The copy numbers of mature *miR-181a* expressed in the DP thymocytes transduced with viral vectors expressing *mir-181a-1*, *mir-181c*, “pre-chimeric”, and “loop-chimeric” miRNAs were determined by miRNA qPCR analyses. Mature *miR-181a* copy numbers in DP cells were determined using standard curve miRNA qPCR quantification and normalized using *miR-15b* as an endogenous control. Representative results of three miRNA qPCR analyses of independently sorted infected DP cells were shown. Statistical significance was determined by an unpaired two-tailed sutdent's t test.
(0.04 MB DOC)

Table S6 Summary of the statistical analyses on the mature *miR-181c* levels in infected DP T cells. The copy numbers of mature *miR-181c* expressed in the DP thymocytes transduced with viral vectors expressing *mir-181a-1*, *mir-181c*, “pre-chimeric”, and “loop-chimeric” miRNAs were determined by miRNA qPCR analyses. Mature *miR-181c* copy numbers in DP cells were determined using standard curve miRNA qPCR quantification and normalized using *miR-15b* as an endogenous control. Representative results of three miRNA qPCR analyses of independently sorted infected DP cells were shown. Statistical significance was determined by an unpaired two-tailed student's t test.
(0.04 MB DOC)

Table S7 Summary of the statistical analyses on the mature *miR-181a* in transfected BOSC cells. The copy numbers of mature *miR-181a* expressed in BOSC cells transfected with constructs expressing *mir-181a-1* loop mutants were determined by quantitative Northern blot. Statistical significance was determined by analyzing the results of four independent quantitative Northern blot analyses using an unpaired two-tailed student's t test.
(0.03 MB DOC)

Table S8 Summary of the statistical analyses on the mature *miR-181a* levels in infected DP T cells. The copy numbers of mature *miR-181a* expressed in the DP thymocytes transduced with viral vectors expressing *mir-181a-1* loop mutants were determined by miRNA qPCR analyses. Mature *miR-181a* copy numbers in DP cells were determined using standard curve miRNA qPCR quantification and normalized using *miR-15b* as an endogenous control. Representative results of three miRNA qPCR analyses of independently sorted infected DP cells were shown. Statistical significance was determined by an unpaired two-tailed student's t test and summarized in the table.
(0.03 MB DOC)
