# Supplementary material for: Pre-miRNA Loop Nucleotides Control the Distinct Activities of mir-181a-1 and mir-181c in Early T Cell Development
Source: PLoS One. 2008 Oct 31;3(10):e3592. doi: 10.1371/journal.pone.0003592 (PMC2575382; doi:10.1371/journal.pone.0003592)
Supplement: Materials and Methods S1 — (0.03 MB DOC) [file pone.0003592.s001.doc]

***Supplemental Information:***

**Supporting Materials and Methods**

**Quantitative Northern blot analyses**

DNA oligos were used as Northern blot probes. Following are the Northern Probe sequences for miR-181a, miR-181c, and stem mutants.

miR-181a ACTCACCGACAGCGTTGAATGTT

miR-181c ACTCACCGACAG GTTGAATGTT

M1 TCACCGACAGCGTTGAATTAT

M2 TCACCGACAGCGTTGATAGTT

M3 TCACCGACAGCGTTTTATGTT

M4 TCACCGACAGCGAAGAATGTT

M5 ACTCACCGACAGGTTTGAATGTT

M6 TCACCGACTTCGTTGAATGTT

M7 ACTCACCGTGAGCGTTGAATGTT

M8 ACTCACGTACAGCGTTGAATGTT

M9 ACTCTGCGACAGCGTTGAATGTT

M10 ACAGACCGACAGCGTTGAATGTT

M11 TGTCACCGACAGCGTTGAATGTT

SM1 ACTCACCGACAGCGTTTTTATAT

SM2 ACTCACCGTGTTGTAAGAATGTT

SM3 TGAGTGGTACAGCGTTGAATGTT

SM4 TGAGTGGTTGTTGTAAGAATGTT

**Primer Extension Analyses**

The extension primers:

15-nt miR-181a primer: 5’ ACTCACCGACAGCGT 3’

15nt miR-181c primer: 5’ ACTCACCGACAGGTT 3’

miR-181a ladder oligos:

16nt: ACTCACCGACAGCGtT

17nt: ACTCACCGACAGCGtTG

18nt: ACTCACCGACAGCGtTGA

19nt: ACTCACCGACAGCGtTGAA

20nt: ACTCACCGACAGCGtTGAAT

21nt: ACTCACCGACAGCGtTGAATG

22nt: ACTCACCGACAGCGtTGAATGT

23nt: ACTCACCGACAGCGtTGAATGTT

miR-181c ladder oligos:

16nt: ACTCACCGACAGGTTG

17nt: ACTCACCGACAGGTTGA

18nt: ACTCACCGACAGGTTGAA

19nt: ACTCACCGACAGGTTGAAT

20nt: ACTCACCGACAGGTTGAATG

21nt: ACTCACCGACAGGTTGAATGT

22nt: ACTCACCGACAGGTTGAATGTT

**Nucleotide sequences of miRNA genes and mutants:**

(1) The nucleotide sequences of wild-type *mir-181a-1* gene

CTCGAGTGTGACAGGTTTGGTTAAAGGATTGGGCTTTCCTCTGCCTCCCTCCTGCTCCAGACTCCCACAGATACTGTTTAAATCAGCACATCTCTGCCTCACAGGTTGCTTCAGTG**AACATTCAACGCTGTCGGTGAGTTTGGAATTCAAATAAAAACCATCGACCGTTGATTGTA**CCCTATAGCTAACCATCATCTACTCCATGGCCCTCTGCGTTTGCTGAAGACAGAACCGCAAAGCAGGACCCGACAGGATTCTTTTTTAATTAAGAATTCCTAGGAATTCTTGCCAAACCTACAGGTGGGGTCTTTCATTCCCCCCTTTTTCTGGAGACTAAATAAAATCTTTTATTTTATCGATAAGCTTGGCTGCAGGTCGACGCGGCCGC

(2) Pre-miRNA nucleotide sequences of the *mir-181a-1* mature and loop mutants

**I. Wild-type pre-miR-181a-1 precursor** AACATTCAACGCTGTCGGTGagtttggaattcaaataaaaACCATCGACCGTTGATTGTA

**II. 2-nt mature miR-181a-1 mutants**

M1: A**ta**ATTCAACGCTGTCGGTGAGTttggaattcaaataaaaACCATCGACCGTTGATT**ta**A

M2: AAC**ta**TCAACGCTGTCGGTGAGTttggaattcaaataaaaACCATCGACCGTTGA**aa**GTA

M3: AACAT**aa**AACGCTGTCGGTGAGTttggaattcaaataaaaACCATCGACCGTT**tt**TTGTA

M4: AACATTC**tt**CGCTGTCGGTGAGTttggaattcaaataaaaACCATCGACCG**aa**GATTGTA

M5: AACATTCAA**ac**CTGTCGGTGAGTttggaattcaaataaaaACCATCGAC**gt**TTGATTGTA

M6: AACATTCAACG**aa**GTCGGTGAGTttggaattcaaataaaaACCATCGACCGTTGATTGTA

M7: AACATTCAACGCT**ca**CGGTGAGTttggaattcaaataaaaACCATCG**tg**CGTTGATTGTA

M8: AACATTCAACGCTGT**ac**GTGAGTttggaattcaaataaaaACCAT**gt**ACCGTTGATTGTA

M9: AACATTCAACGCTGTCG**ca**GAGTttggaattcaaataaaaACC**tg**CGACCGTTGATTGTA

M10: AACATTCAACGCTGTCGGT**ct**GTttggaattcaaataaaaAC**g**ATCGACCGTTGATTGTA

M11: AACATTCAACGCTGTCGGTGA**ca**ttggaattcaaataaaa**tg**CATCGACCGTTGATTGTA

**III. Segment mature *miR-181a-1* mutants**

(1)SM1: A**tataaa**AACGCTGTCGGTGAGTttggaattcaaataaaaACCATCGACCGTT**tttata**A

(2)SM2: AACATTC**ttacaaca**CGGTGAGTttggaattcaaataaaaACCATCG**tggtaa**GATTGTA

(3)SM3: AACATTCAACGCTGT**accactca**ttggaattcaaataaaa**tggtggt**ACCGTTGATTGTA

(4)SM4: AACATTC**ttacaacaaccactca**ttggaattcaaataaaa**tggtggttggtaa**GATTGTA

**IV. *miR-181a-1* Loop Mutant**

**(1)** **181a-LP1:** AACATTCAACGCTGTCGGTGAGTtt**CC**aattcaaataaaaACCATCGACCGTTGATTGTA

(2) **181a-LP2:** AACATTCAACGCTGTCGGTGAGTttgg**TT**ttcaaataaaaACCATCGACCGTTGATTGTA

(3) **181a-LP3:** AACATTCAACGCTGTCGGTGAGTttggaa**AA**caaataaaaACCATCGACCGTTGATTGTA

(4) **181a-LP4:** AACATTCAACGCTGTCGGTGAGTttggaatt**GT**aataaaaACCATCGACCGTTGATTGTA

**(5)** **181a-LP5:** AACATTCAACGCTGTCGGTGAGTttggaattca**CG**taaaaACCATCGACCGTTGATTGTA

(6) **181a-LP6:** AACATTCAACGCTGTCGGTGAGTttggaattcaaa**AT**aaaACCATCGACCGTTGATTGTA

(3) The nucleotide sequences of the wild-type *mir-181c* gene

GATCCTCGAGGTGAGTCAAAGGGGACCCTGGTTTCTCTCTCGTCCCACATGCTCTCTGCCTTGCTGGCCTACTCTCCCAACTCCAGTTATCCAAGAACTTGCCAAGGGTTTGGGGG**AACATTCAACCTGTCGGTGAGTTTGGGCAGCTCAGACAAACCATCGACCGTTGAGTGGA**CCCCGAGGCCTGGAACTGCCACCCGTCTACCCCATCCCCACCCTGTAGACCCGGGAGAGCCCCAGGCAGCATCCCctgcctcaggccacagcaaaggtcacaattGAATTCGATC
