# Supplementary figures and images for: Pre-miRNA Loop Nucleotides Control the Distinct Activities of mir-181a-1 and mir-181c in Early T Cell Development
Source: PLoS One. 2008 Oct 31;3(10):e3592. doi: 10.1371/journal.pone.0003592 (PMC2575382; doi:10.1371/journal.pone.0003592)

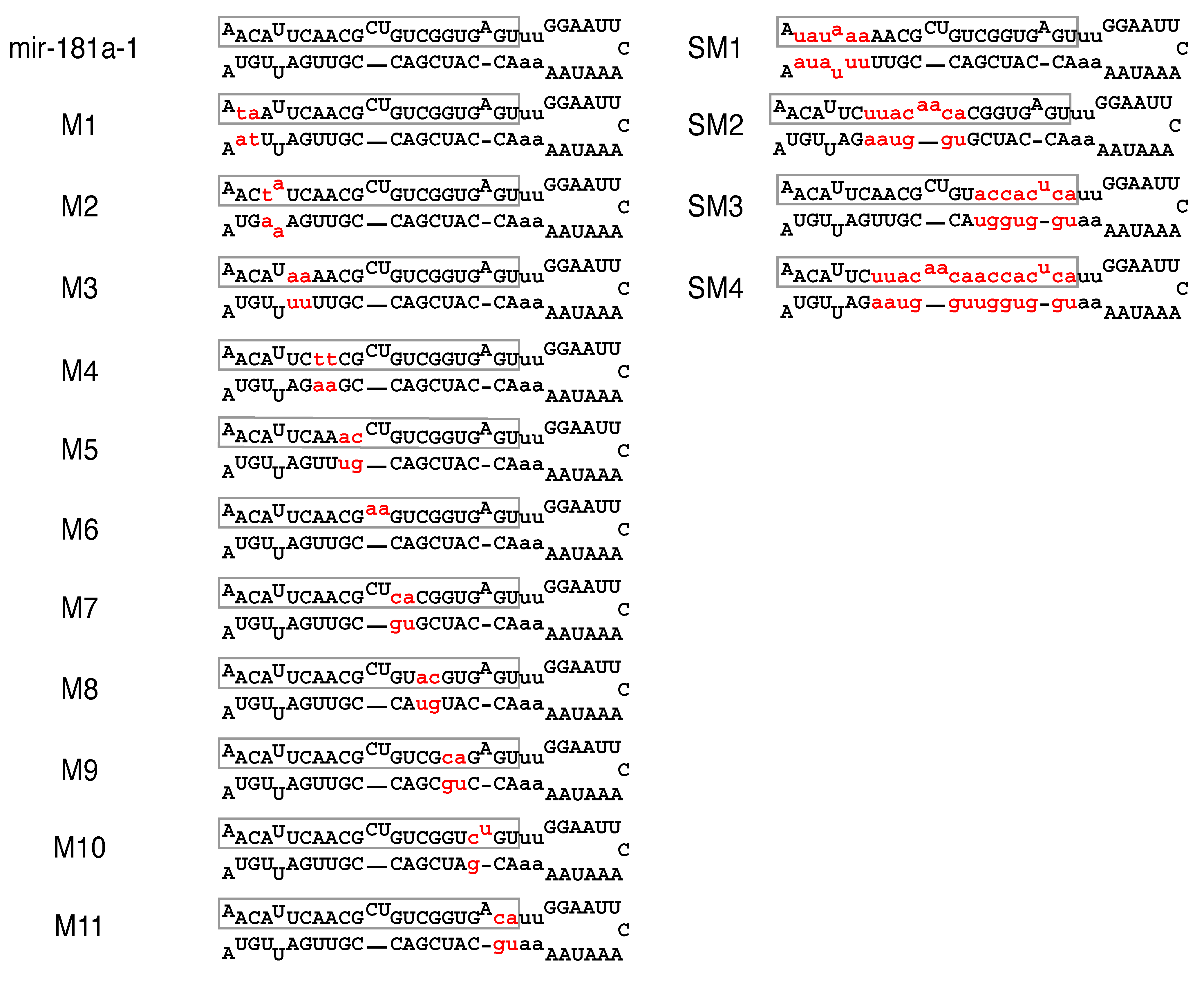

Supplement: Figure S1 — Schematics and nucleotide sequences depict mature mir-181a-1 mutants. Compensatory mutations are introduced to maintain the integrity of the pre-miRNA secondary structure. (0.95 MB TIF) [file pone.0003592.s002.tif]

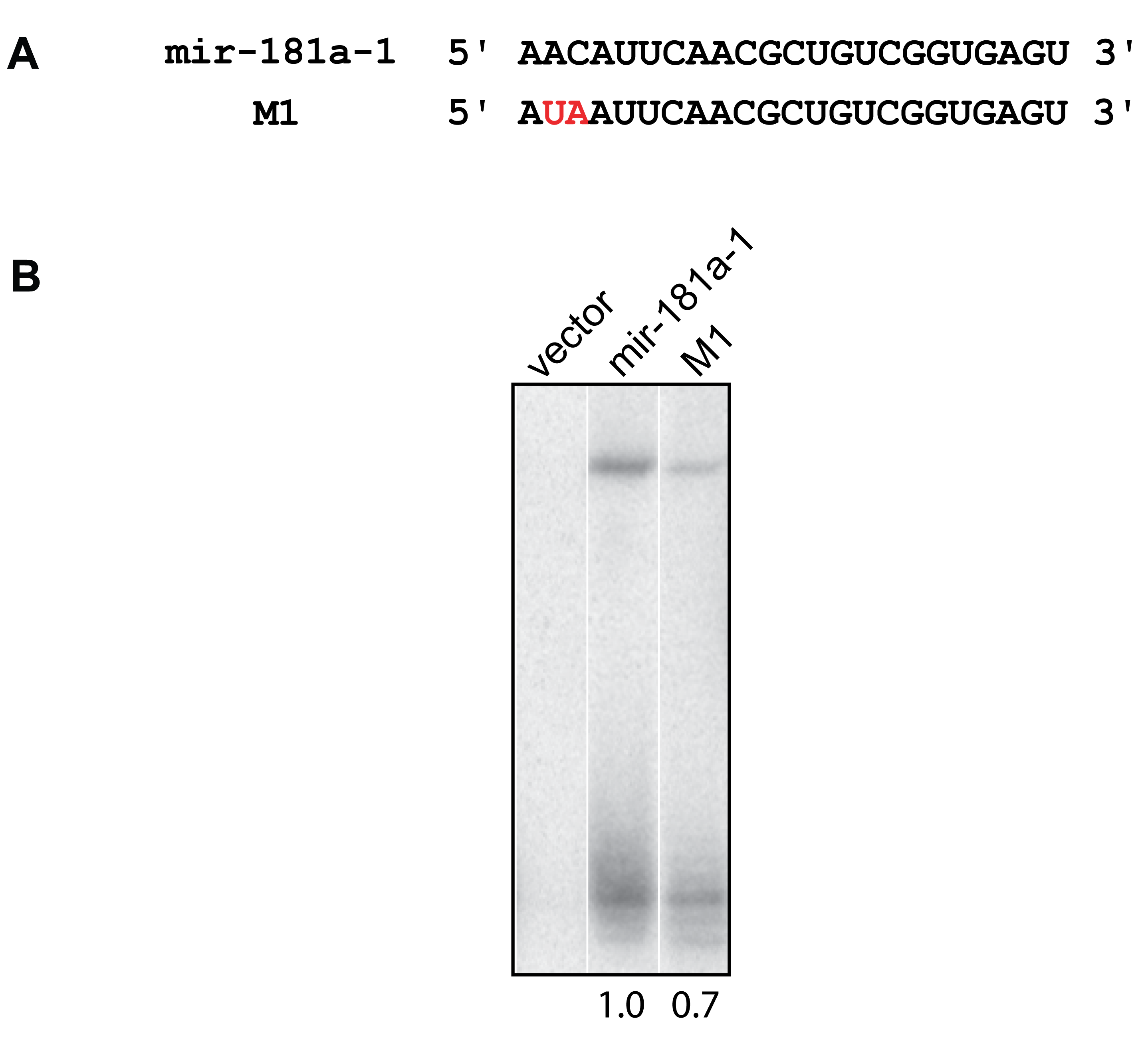

Supplement: Figure S2 — Expression and processing of wild-type mir-181a-1 and the M1 stem mutant gene. (A) Nucleotide sequences of the wild-type miR-181a and the M1 mutant. (B) Northern blot analyses of mature miRNA expression from the wild-type miR-181a and the M1 mutant. Total RNA was prepared from BOSC cells transfected with constructs expressing mir-181a-1, or the M1 mutant genes. Relative transfection efficiencies were determined by qPCR analyses of GFP mRNA levels produced from the transfected miRNA constructs, then used to normalize RNA loadings in Northern blot analyses. A shorter probe that perfectly matches to both mature miR-181a and the M1 mutant forms is used in hybridization to determine the expression of mature miR-181a and its mutant forms. Relative expression levels of the mature miRNAs determined by phosphoimager quantification is indicated. (1.46 MB TIF) [file pone.0003592.s003.tif]

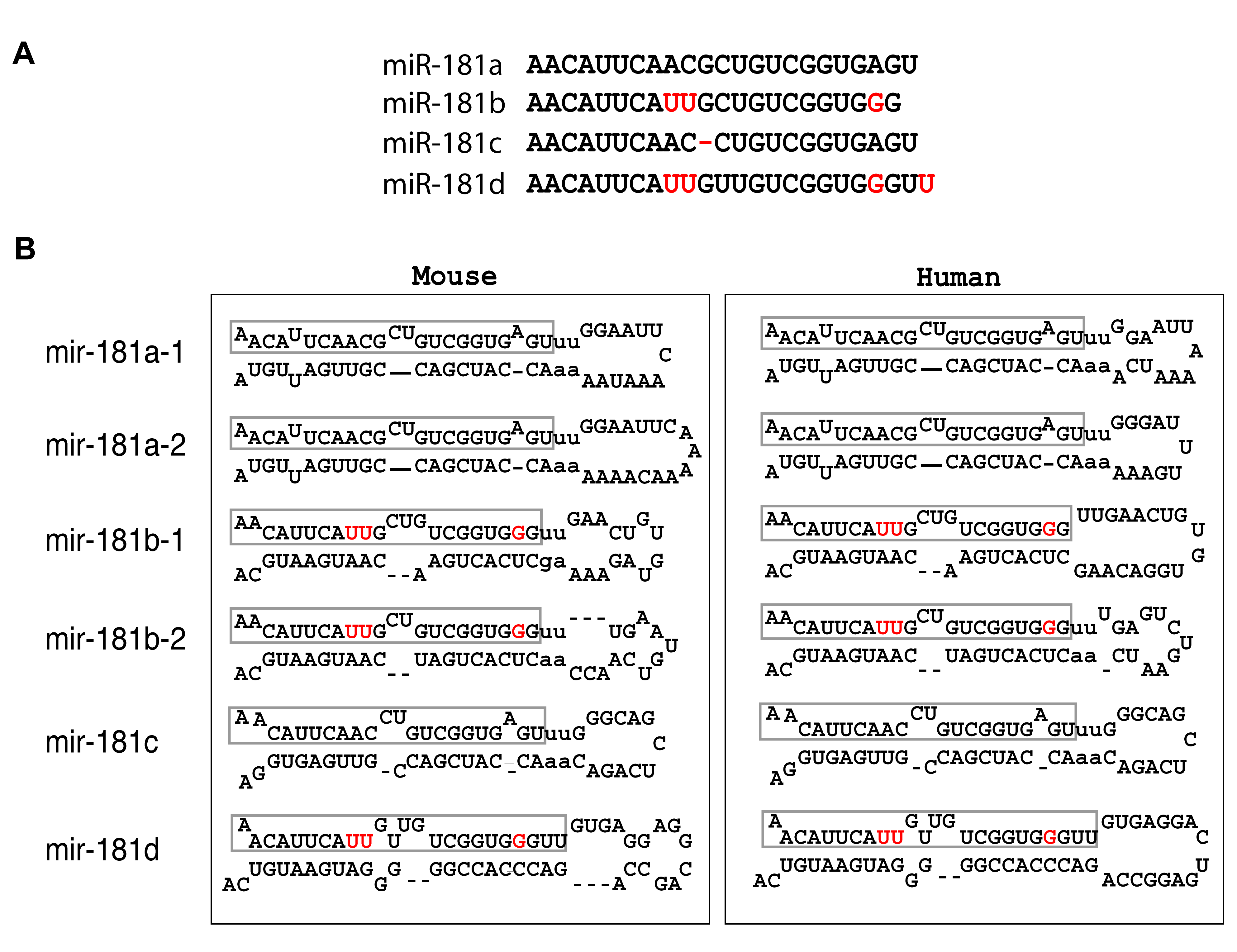

Supplement: Figure S3 — Members of the mir-181 gene family. (A) Alignment of the mature miR-181 miRNAs. (B) Schematics and nucleotide sequences depicting the pre-miRNAs of the human and mouse mir-181 gene family members. (0.98 MB TIF) [file pone.0003592.s004.tif]

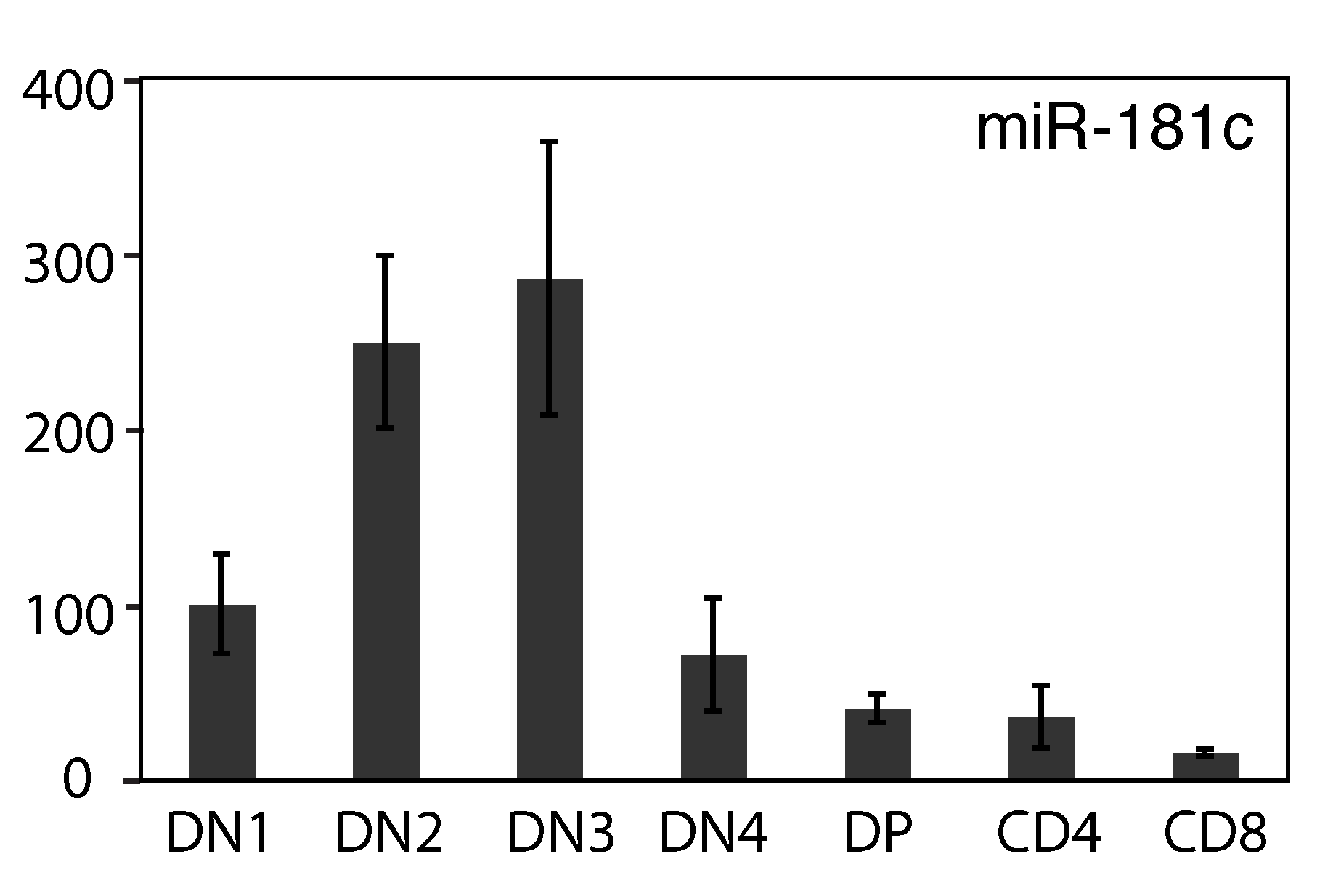

Supplement: Figure S4 — Developmental regulation of miR-181c expression in various purified thymocyte populations determined by miRNA qPCR. (0.23 MB TIF) [file pone.0003592.s005.tif]

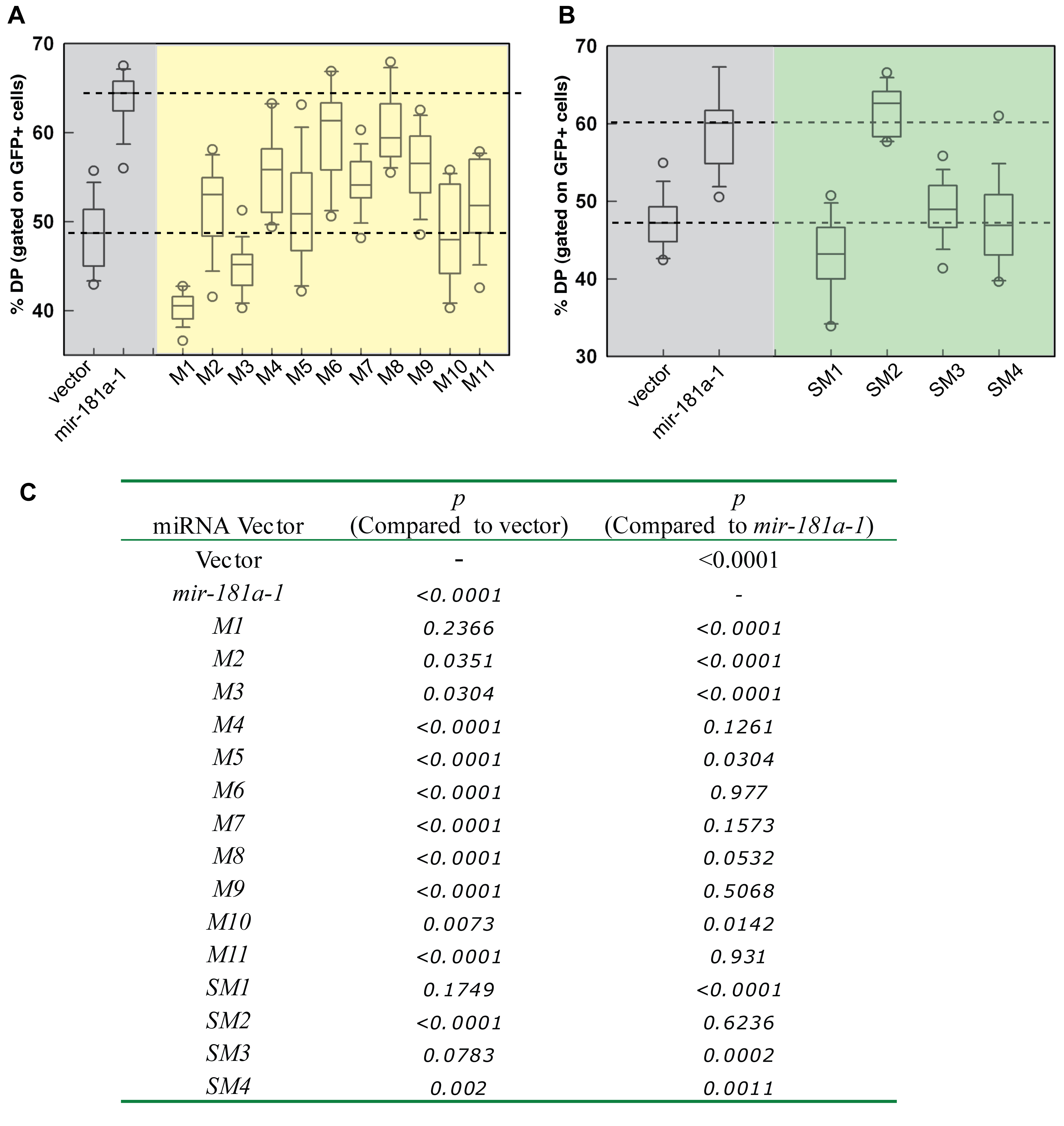

Supplement: Figure S5 — The effects of mutations in the mature miRNA region of the mir-181a-1 genes on DP cell development (SI Fig. 1). (A) Box-plots summarize the percent of DP cells generated from DN progenitor cells infected with mir-181a-1, or mature miRNA mutant genes (gated on GFP positive). A representative OP9-DL1 stromal co-culture assay (12 independent replicates for each construct) is shown. The ends of the boxes define the 25th and 75th percentiles, a line indicates the median, and bars define the 5th and 95th percentiles. (B) Statistical summary. Mann-Whitney Rank Sum Tests were performed on this representative data set to determine whether the activity of mir-181a-1, mir-181c, or their chimeric mutants is statistically different from the control vector or the mir-181a-1 vector. (2.34 MB TIF) [file pone.0003592.s006.tif]

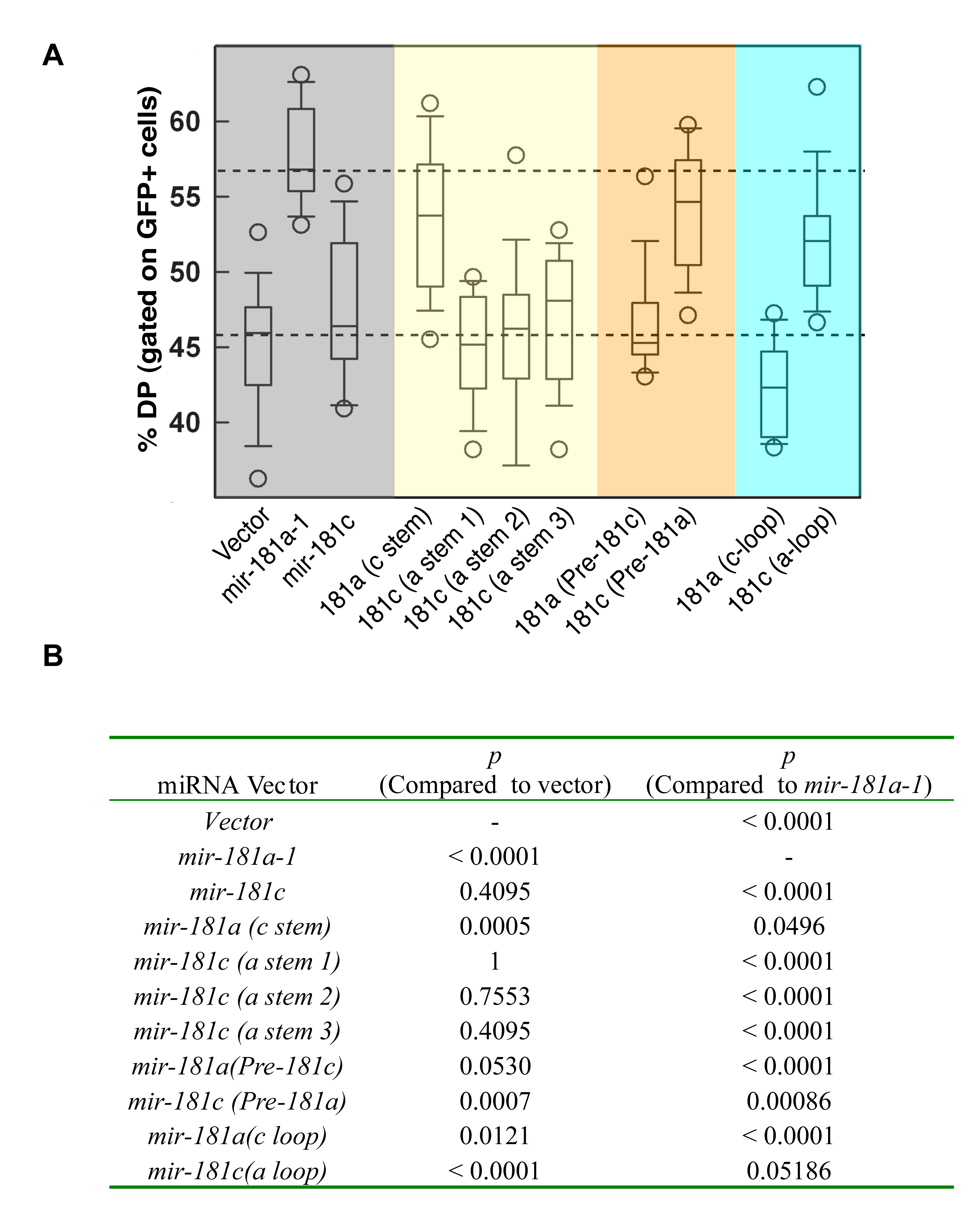

Supplement: Figure S6 — The effects of the chimeric mir-181a-1 and mir-181c genes on DP cell development (Fig. 2). (A) Box-plots summarize the percent of DP cells generated from DN progenitor cells infected with mir-181a-1, mir-181c, or their chimeric mutants (GFP positive). A representative OP9-DL1 stromal co-culture assay (12 independent replicates for each construct) is shown. The ends of the boxes define the 25th and 75th percentiles, a line indicates the median, and bars define the 5th and 95th percentiles. (B) Statistical summary. Mann-Whitney Rank Sum Tests were performed on this representative data set to determine whether the activity of mir-181a-1, mir-181c, or their chimeric mutants is statistically different from the control vector or the mir-181a-1 vector. (1.92 MB TIF) [file pone.0003592.s007.tif]

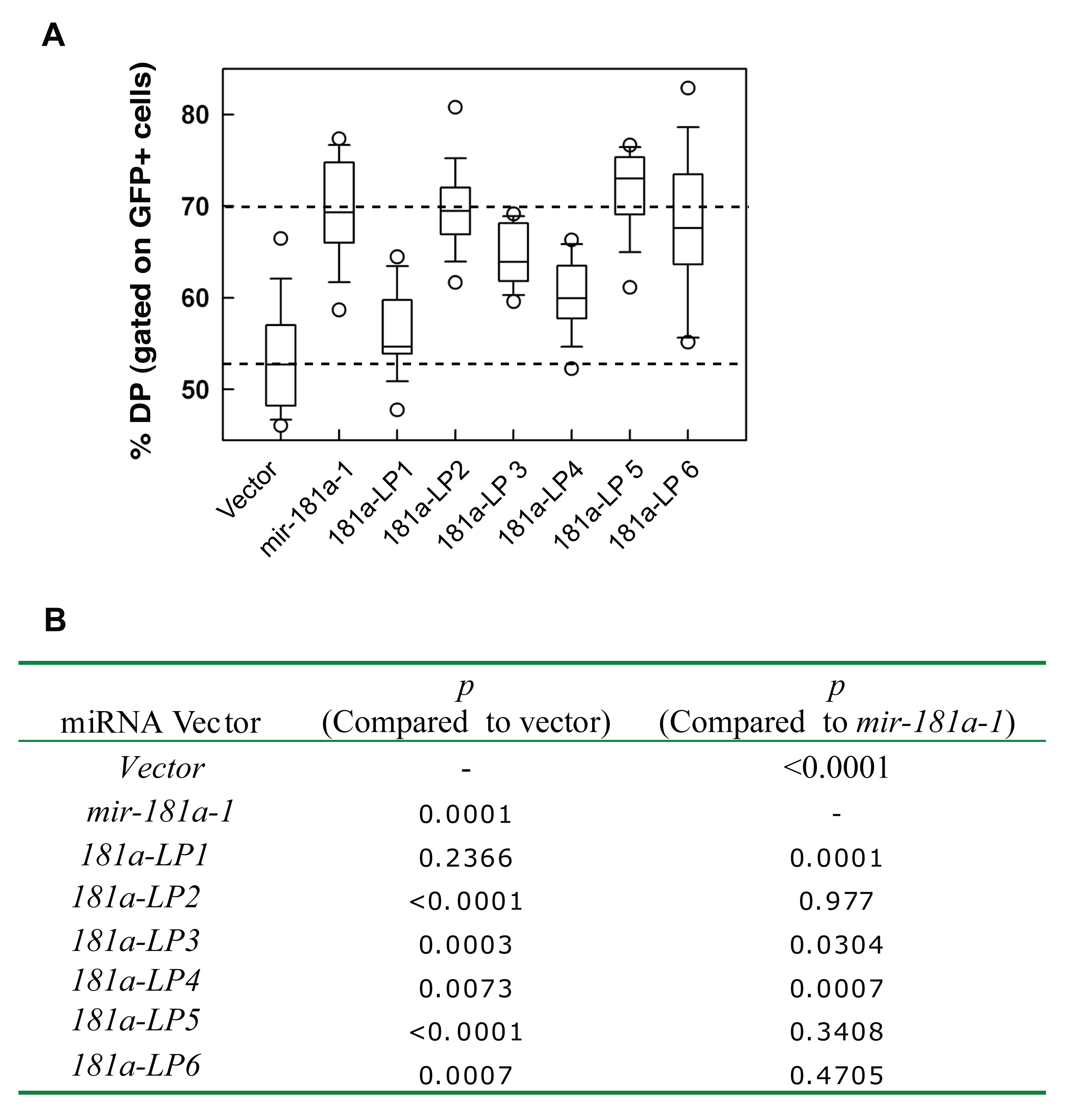

Supplement: Figure S7 — The effects of the pre-miR-181a-1 loop mutants on DP cell development (Fig. 3). (A) Box-plots summarize the percent of DP cells generated from DN progenitor cells infected with mir-181a-1, or pre-miR-181a-1 loop mutant genes (GFP positive). A representative OP9-DL1 stromal co-culture assay (12 independent replicates for each construct) is shown. The ends of the boxes define the 25th and 75th percentiles, a line indicates the median, and bars define the 5th and 95th percentiles. (B) Statistical summary. Mann-Whitney Rank Sum Tests are performed on this representative data set to determine whether the activity of mir-181a-1 and pre-miRNA loop mutant genes is statistically different from the empty vector (negative control) or the mir-181a-1 expressing vector (positive control). (1.07 MB TIF) [file pone.0003592.s008.tif]

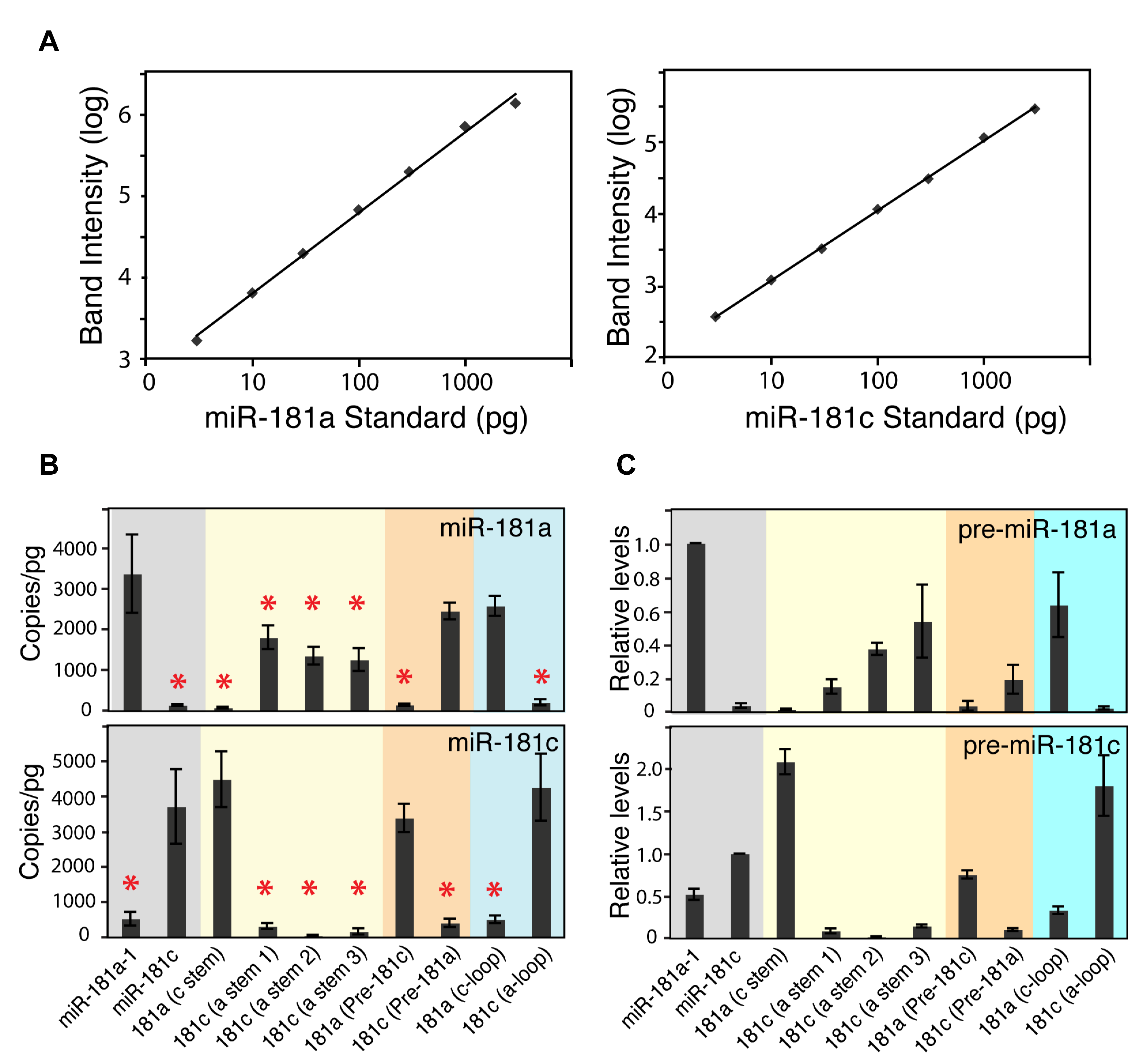

Supplement: Figure S8 — Mature and pre-miRNA expression levels from the chimeric mir-181a-1 and mir-181c genes (Fig. 5A, B). Total RNA was prepared from BOSC cells transfected with constructs expressing mir-181a-1, mir-181c, and the chimeric mir-181a-1 and mir-181c genes. Since all miRNA vectors contain an independent GFP reporter, percentage cells that are GFP positive were determined by FACS analyses and used to control for variations in transfection efficiency. Quantitative Northern blot analyses were carried out to determine the expression of mir-181a-1, mir-181c, and the chimeric mir-181a-1 and mir-181c genes. Specific probes that perfectly match to mature miR-181a or miR-181c were used in hybridization to determine the expression of mature and pre-miRNA forms. Band intensities were determined by phosphoimager quantification and normalized to the levels of wild-type controls accordingly. (A) Standard Curves for miR-181a and miR-181c. (B) The copies of mature miR-181a and miR-181c in BOSC 23 cells transfected with mir-181a-1/c mutants determined by quantitative Northern blot analyses. Average results of four independent experiments were plotted ( Table S4 for statistics). (C) Relative levels of pre-miR-181a and pre-miR-181c in BOSC 23 cells transfected with mir-181a-1/c mutants determined by Northern blot analyses. Average results of four independent experiments were plotted. (2.36 MB TIF) [file pone.0003592.s009.tif]

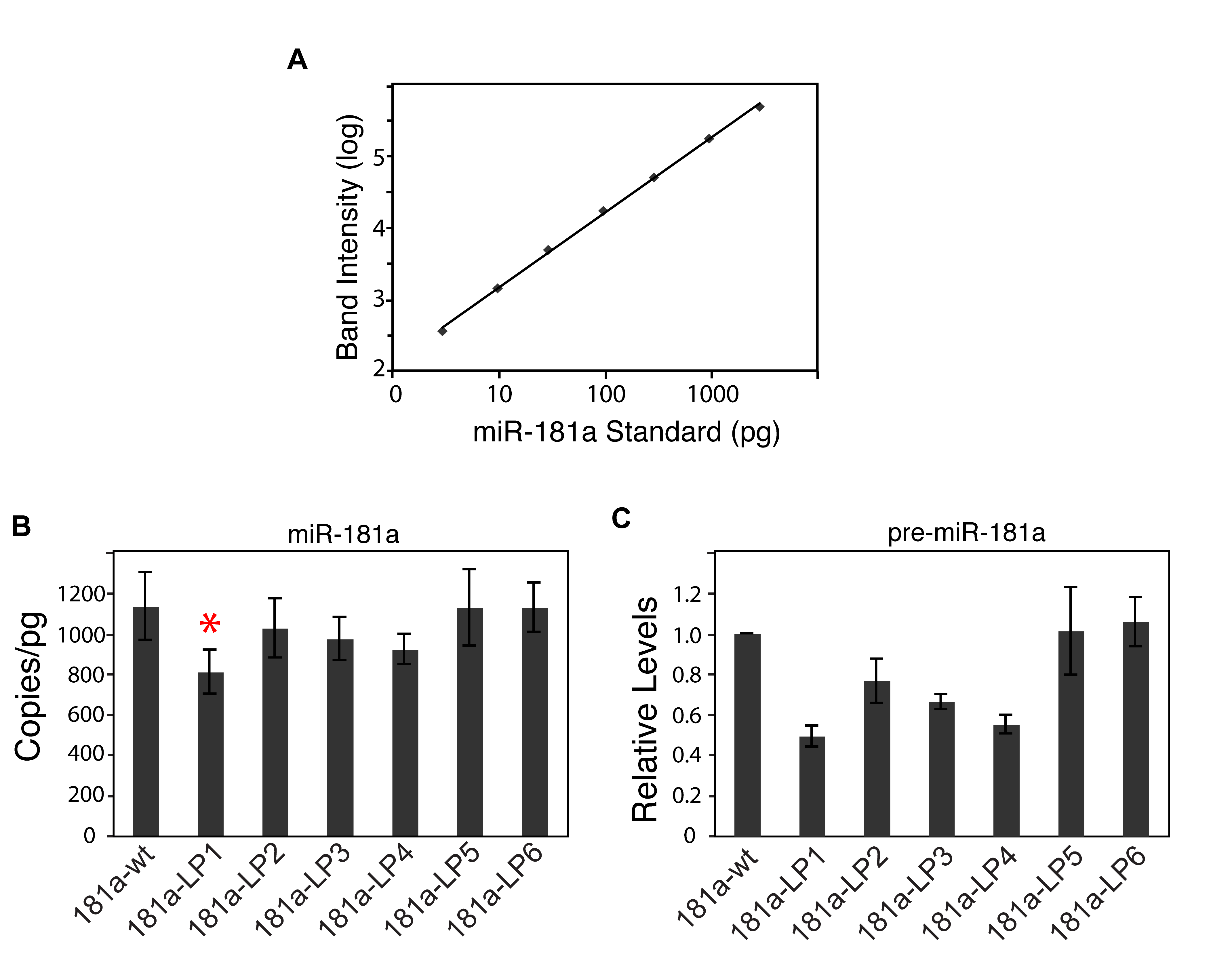

Supplement: Figure S9 — Mature and pre-miRNA expression levels from the pre-miR-181a-1 loop mutant genes (Fig. 5E). Total RNA was prepared from BOSC cells transfected with constructs expressing the mir-181a-1 loop mutant genes. Since all miRNA vectors contain an independent GFP reporter, percentage cells that are GFP positive were determined by FACS analyses and used to control for variations in transfection efficiency. Quantitative Northern blot analyses were carried out to determine the expression of the pre-mir-181a-1 loop mutant genes. A probe that perfectly matches to the mature miR-181a was used in hybridization to determine the expression of mature and pre-miRNA forms. Band intensity was determined by phosphoimager quantification and normalized to the levels of wild-type controls accordingly. (A) Standard Curves for miR-181a. (B) The copies of mature miR-181a in BOSC 23 cells transfected with mir-181a-1 loop mutants determined by quantitative Northern blot analyses. Average results of four independent experiments were plotted ( Table S7 for statistics). (C) Relative levels of pre-miR-181a in BOSC 23 cells transfected with mir-181a-1 loop mutants determined by Northern blot analyses. Average results of four independent experiments were plotted. (0.92 MB TIF) [file pone.0003592.s010.tif]
