# Supplementary material for: Pre-miRNA Loop Nucleotides Control the Distinct Activities of mir-181a-1 and mir-181c in Early T Cell Development
Source: PLoS One. 2008 Oct 31;3(10):e3592. doi: 10.1371/journal.pone.0003592 (PMC2575382; doi:10.1371/journal.pone.0003592)
Supplement: Table S1 — Summary of the statistical analyses on the activity of mir-181a-1 genes with mutations in the mature miRNA region. The activities of the wild-type mir-181a-1 and its variants with mutations in the mature miRNA regions in promoting DP cell development are normalized so that the empty vector (negative control) has a median activity of “0” and the wild-type mir-181a-1 vector (positive control) has a median activity of “1.” Normalized data from 3–5 independent T cell assays (each with 12 independent replicates, total 36–60 replicates) are pooled and graphed in the distribution box plots. Mann-Whitney Rank Sum Tests are performed to determine whether the activity of mir-181a-1 and mature miRNA mutant genes is statistically different from the empty vector (negative control) or the mir-181a-1 expressing vector (positive control). (0.05 MB DOC) [file pone.0003592.s011.doc]

| miRNA Vector | n  (no. of replicates) | *p*  (Compared to vector) | *p*  (Compared to *mir-181a-1*) |
| --- | --- | --- | --- |
| Vector | 60 | - | <0.0001 |
| *mir-181a-1* | 60 | <0.0001 | - |
| *M1* | 56 | 0.0004 | <0.0001 |
| *M2* | 57 | 0.0011 | <0.0001 |
| *M3* | 60 | 0.0674 | <0.0001 |
| *M4* | 60 | <0.0001 | <0.0001 |
| *M5* | 60 | <0.0001 | <0.0001 |
| *M6* | 60 | <0.0001 | 0.1453 |
| *M7* | 60 | <0.0001 | 0.0033 |
| *M8* | 60 | <0.0001 | 0.0071 |
| *M9* | 60 | <0.0001 | 0.1083 |
| *M10* | 48 | <0.0001 | <0.0001 |
| *M11* | 36 | <0.0001 | 0.0124 |
| *SM1* | 40 | 0.9807 | <0.0001 |
| *SM2* | 48 | <0.0001 | 0.9679 |
| *SM3* | 48 | 0.0004 | <0.0001 |
| *SM4* | 42 | 0.0001 | <0.0001 |
