# Supplementary material for: Pre-miRNA Loop Nucleotides Control the Distinct Activities of mir-181a-1 and mir-181c in Early T Cell Development
Source: PLoS One. 2008 Oct 31;3(10):e3592. doi: 10.1371/journal.pone.0003592 (PMC2575382; doi:10.1371/journal.pone.0003592)
Supplement: Table S2 — Summary of the statistical analyses on the activity of the mir-181a-1 and mir-181c mutant genes. The activities of mir-181a-1, mir-181c, and mutant genes in promoting DP cell development are normalized so that the empty vector (negative control) has a median activity of “0” and the mir-181a-1 expressing vector (positive control) has a median activity of “1.” Normalized data from 3–7 independent T cell assays (each with 12 independent replicates, total 36–84 replicates) are pooled and graphed in the distribution box plots. Mann-Whitney Rank Sum Tests are performed on the pooled data set to determine whether the activity of mir-181a-1 and mir-181c mutant genes is statistically different from the empty vector (negative control) or the mir-181a-1 expressing vector (positive control). (0.04 MB DOC) [file pone.0003592.s012.doc]

| miRNA Vector | n  (no of replicates) | *p*  (Compared to vector) | *p*  (Compared to *mir-181a-1*) |
| --- | --- | --- | --- |
| Vector | 84 | - | < 0.0001 |
| *mir-181a-1* | 84 | < 0.0001 | - |
| *mir-181c* | 84 | 0.092 | < 0.0001 |
| *mir-181a (c stem)* | 60 | < 0.0001 | < 0.0001 |
| *mir-181c (a stem 1)* | 60 | <0.0001 | < 0.0001 |
| *mir-181c (a stem 2)* | 36 | 0.699 | < 0.0001 |
| *mir-181c (a stem 3)* | 36 | 0.335 | < 0.0001 |
| *mir-181a(Pre-181c)* | 36 | 0.494 | < 0.0001 |
| *mir-181c (Pre-181a)* | 36 | < 0.0001 | < 0.0001 |
| *mir-181a(c loop)* | 36 | 0.057 | < 0.0001 |
| *mir-181c(a loop)* | 36 | < 0.0001 | < 0.0001 |
