# Supplementary material for: Pre-miRNA Loop Nucleotides Control the Distinct Activities of mir-181a-1 and mir-181c in Early T Cell Development
Source: PLoS One. 2008 Oct 31;3(10):e3592. doi: 10.1371/journal.pone.0003592 (PMC2575382; doi:10.1371/journal.pone.0003592)
Supplement: Table S3 — Summary of the statistical analyses on the activity of the mir-181a-1 genes and mutants with nucleotides in the pre-miRNA loop region altered. The activity of mir-181a-1 and its pre-miRNA loop mutant genes in promoting DP cell development are normalized so that the empty vector (negative control) has a median activity of “0” and the mir-181a-1 expressing vector (positive control) has a median activity of “1.” Normalized data from at least 6 independent T cell assays (each with 12 independent replicates, total 72 replicates) are pooled and graphed in the distribution box plots. Mann-Whitney Rank Sum Tests are performed on the pooled data set to determine whether the activity of mir-181a-1 and pre-miRNA loop mutant genes is statistically different from the empty vector (negative control) or the mir-181a-1 expressing vector (positive control). (0.03 MB DOC) [file pone.0003592.s013.doc]

| miRNA Vector | n  (no of replicates) | *p*  (Compared to vector) | *p*  (Compared to *mir-181a-1*) |
| --- | --- | --- | --- |
| Vector | 72 | - | <0.0001 |
| *mir-181a-1* | 72 | <0.0001 | - |
| *181a-LP1* | 72 | <0.0001 | <0.0001 |
| *181a-LP2* | 72 | <0.0001 | 0.6035 |
| *181a-LP3* | 72 | <0.0001 | <0.0001 |
| *181a-LP4* | 72 | <0.0001 | <0.0001 |
| *181a-LP5* | 72 | <0.0001 | 0.8841 |
| *181a-LP6* | 72 | <0.0001 | 0.9474 |
