# Supplementary material for: Pre-miRNA Loop Nucleotides Control the Distinct Activities of mir-181a-1 and mir-181c in Early T Cell Development
Source: PLoS One. 2008 Oct 31;3(10):e3592. doi: 10.1371/journal.pone.0003592 (PMC2575382; doi:10.1371/journal.pone.0003592)
Supplement: Table S4 — Summary of the statistical analyses on the mature miR-181a and miR-181c levels in transfected BOSC cells. The copy numbers of mature miR-181a and miR-181c expressed in BOSC cells transfected with constructs expressing mir-181a-1, mir-181c, and the chimeric mir-181a-1 and mir-181c genes were determined by quantitative Northern blot. Statistical significance was determined by analyzing the results of four independent quantitative Northern blot analyses using an unpaired two-tailed student's t test. (0.03 MB DOC) [file pone.0003592.s014.doc]

| miRNA Vector | *p*  (Compared to *mir-181a-1* ) | | *p*  (Compared to *mir-181c*) |
| --- | --- | --- | --- |
| *mir-181a-1* | - | 0.006 | |
| *mir-181c* | 0.0005 | - | |
| *mir-181a (c stem)* | 0.0005 | 0.3508 | |
| *mir-181c (a stem 1)* | 0.0221 | 0.0049 | |
| *mir-181c (a stem 2)* | 0.0067 | 0.0039 | |
| *mir-181c (a stem 3)* | 0.006 | 0.0046 | |
| *mir-181a(Pre-181c)* | 0.0005 | 0.7735 | |
| *mir-181c (Pre-181a)* | 0.1138 | 0.0055 | |
| *mir-181a(c-loop)* | 0.166 | 0.0062 | |
| *mir-181c(a-loop)* | 0.0006 | 0.3415 | |
