# Supplementary material for: Pre-miRNA Loop Nucleotides Control the Distinct Activities of mir-181a-1 and mir-181c in Early T Cell Development
Source: PLoS One. 2008 Oct 31;3(10):e3592. doi: 10.1371/journal.pone.0003592 (PMC2575382; doi:10.1371/journal.pone.0003592)
Supplement: Table S5 — Summary of the statistical analyses on the mature miR-181a levels in infected DP T cells. The copy numbers of mature miR-181a expressed in the DP thymocytes transduced with viral vectors expressing mir-181a-1, mir-181c, “pre-chimeric”, and “loop-chimeric” miRNAs were determined by miRNA qPCR analyses. Mature miR-181a copy numbers in DP cells were determined using standard curve miRNA qPCR quantification and normalized using miR-15b as an endogenous control. Representative results of three miRNA qPCR analyses of independently sorted infected DP cells were shown. Statistical significance was determined by an unpaired two-tailed sutdent's t test. (0.04 MB DOC) [file pone.0003592.s015.doc]

| miRNA Vector | *p*  (Compared to vector) | *p*  (Compared to *mir-181a-1*) |
| --- | --- | --- |
| Vector | - | - |
| *mir-181a-1* | 0.1337 | - |
| *mir-181c* | 0.002 | 0.0004 |
| *mir-181a (c stem)* | 0.0021 | 0.0004 |
| *mir-181c (a stem 1)* | 0.2417 | 0.075 |
| *mir-181c (a stem 2)* | 0.4831 | 0.1006 |
| *mir-181c (a stem 3)* | 0.0597 | 0.4477 |
| *mir-181a(Pre-181c)* | 0.1585 | 0.0208 |
| *mir-181c (Pre-181a)* | 0.068 | 0.0977 |
| *mir-181a(c-loop)* | 0.0528 | 0.0175 |
| *mir-181c(a-loop)* | 0.0403 | 0.0042 |
