# Supplementary material for: Pre-miRNA Loop Nucleotides Control the Distinct Activities of mir-181a-1 and mir-181c in Early T Cell Development
Source: PLoS One. 2008 Oct 31;3(10):e3592. doi: 10.1371/journal.pone.0003592 (PMC2575382; doi:10.1371/journal.pone.0003592)
Supplement: Table S6 — Summary of the statistical analyses on the mature miR-181c levels in infected DP T cells. The copy numbers of mature miR-181c expressed in the DP thymocytes transduced with viral vectors expressing mir-181a-1, mir-181c, “pre-chimeric”, and “loop-chimeric” miRNAs were determined by miRNA qPCR analyses. Mature miR-181c copy numbers in DP cells were determined using standard curve miRNA qPCR quantification and normalized using miR-15b as an endogenous control. Representative results of three miRNA qPCR analyses of independently sorted infected DP cells were shown. Statistical significance was determined by an unpaired two-tailed student's t test. (0.04 MB DOC) [file pone.0003592.s016.doc]

| miRNA Vector | *p*  (Compared to vector) | *p*  (Compared to *mir-181c*) |
| --- | --- | --- |
| Vector | - | - |
| *mir-181a-1* | 0.2003 | 1 |
| *mir-181c* | 0.2003 | - |
| *mir-181a (c stem)* | 0.0152 | 0.0122 |
| *mir-181c (a stem 1)* | 0.5799 | 0.3647 |
| *mir-181c (a stem 2)* | 0.0006 | 0.2314 |
| *mir-181c (a stem 3)* | 0.8869 | 0.647 |
| *mir-181a(Pre-181c)* | 0.0408 | 0.0278 |
| *mir-181c (Pre-181a)* | 0.0068 | 0.0061 |
| *mir-181a(c-loop)* | 0.7037 | 0.5199 |
| *mir-181c(a-loop)* | 0.0138 | 0.011 |
