# Supplementary material for: Pre-miRNA Loop Nucleotides Control the Distinct Activities of mir-181a-1 and mir-181c in Early T Cell Development
Source: PLoS One. 2008 Oct 31;3(10):e3592. doi: 10.1371/journal.pone.0003592 (PMC2575382; doi:10.1371/journal.pone.0003592)
Supplement: Table S7 — Summary of the statistical analyses on the mature miR-181a in transfected BOSC cells. The copy numbers of mature miR-181a expressed in BOSC cells transfected with constructs expressing mir-181a-1 loop mutants were determined by quantitative Northern blot. Statistical significance was determined by analyzing the results of four independent quantitative Northern blot analyses using an unpaired two-tailed student's t test. (0.03 MB DOC) [file pone.0003592.s017.doc]

| miRNA Vector | *p*  (*Compared to mir-181a-1*) |
| --- | --- |
| *mir-181a-1* | - |
| *181a-LP1* | 0.0177 |
| *181a-LP2* | 0.3676 |
| *181a-LP3* | 0.1559 |
| *181a-LP4* | 0.0599 |
| *181a-LP5* | 0.9588 |
| *181a-LP6* | 0.9573 |
|  |  |
