# Supplementary material for: Pre-miRNA Loop Nucleotides Control the Distinct Activities of mir-181a-1 and mir-181c in Early T Cell Development
Source: PLoS One. 2008 Oct 31;3(10):e3592. doi: 10.1371/journal.pone.0003592 (PMC2575382; doi:10.1371/journal.pone.0003592)
Supplement: Table S8 — Summary of the statistical analyses on the mature miR-181a levels in infected DP T cells. The copy numbers of mature miR-181a expressed in the DP thymocytes transduced with viral vectors expressing mir-181a-1 loop mutants were determined by miRNA qPCR analyses. Mature miR-181a copy numbers in DP cells were determined using standard curve miRNA qPCR quantification and normalized using miR-15b as an endogenous control. Representative results of three miRNA qPCR analyses of independently sorted infected DP cells were shown. Statistical significance was determined by an unpaired two-tailed student's t test and summarized in the table. (0.03 MB DOC) [file pone.0003592.s018.doc]

| miRNA Vector | *p*  (Compared to vector) | *p*  (Compared to *mir-181a*) |
| --- | --- | --- |
| Vector | - | - |
| *mir-181a-1* | 0.1752 | - |
| *181a-LP1* | 0.0375 | 0.2143 |
| *181a-LP2* | 0.0044 | 0.0022 |
| *181a-LP3* | 0.0336 | 0.063 |
| *181a-LP4* | 0.0149 | 0.0192 |
| *181a-LP5* | 0.0131 | 0.0176 |
| *181a-LP6* | 0.1383 | 0.7813 |
